# Addiction spectrum disorder: a conceptual framework for comprehensive understanding of addictive disorders

**DOI:** 10.3389/fpsyg.2026.1753662

**Published:** 2026-02-27

**Authors:** Yukiori Goto, Jana Krieger, Moojun Won, Young-A Lee

**Affiliations:** 1 School of Informatics, Kyoto University, Kyoto, Japan; 2 Neuroscience Program, Rheinische Friedrich-Wilhelms Universität, Bonn, Germany; 3 MRC Lab Clinic, Mitaka, Japan; 4 Department of Food Science and Nutrition, Daegu Catholic University, Gyeongsan, Gyeongbuk, Republic of Korea

**Keywords:** associative learning, behavioral addiction, eating disorder, food addiction, impulse control disorder, obsessive compulsive disorder, negative affect, substance use disorder

## Abstract

The term addiction or addictive disorder refers to a psychiatric condition that is characterized impulsive and compulsive seeking targets or action executions despite negative consequences are expected to occur. However, it has been used to lump widely heterogeneous conditions, such as substance use disorders, behavioral addictions, and food addiction, together, which has been causing a serious problem in understanding and defining the addictive disorder. Here we deliberate a framework toward comprehensive understanding of addictive disorders that overcome the heterogeneities of substance use disorders, behavioral addictions, and a food addiction, by considering that addictive disorders could form a spectrum of disorders, consisting of three imperative components, specifically negative reinforcements in relation to compulsive seeking, cue-induced responses in relation to associative learning, and a food addiction as an intermediate phenotype between substance use disorders and behavioral addictions.

## 1 Introduction

The term “addiction (or addictive disorder)” has been used to refer to mental health conditions in the general public as well as in scientific and clinical fields. However, what does “addiction” really mean? Addiction was used to be synonymous with drug addiction or substance use disorder (SUD); however, this has recently been extended to include addictions to specific actions, i.e., behavioral addiction (BA) and food addiction (FA). Although FA is not yet an independent diagnosis in the International Classification of Diseases, 10th revision (ICD-10; World Health Organization, 1992) as well as the Diagnostic and Statistical Manual of Mental Disorders, 5th version (DSM-5; American Psychiatric Association, 2013), it has been discussed in neuroscience literature for addictive disorder as a possible subtype, with a particular focus on addiction-related behaviors (Vasiliu, 2021). Studies indicate that while substance and non-substance addictions exhibit symptomatic overlaps, the proposed diagnostic criteria for many non-substance addictions, including eating disorders, are currently not sufficiently clinically validated and primarily serve as research tools (Wei and Zhang, 2017). For this reason, we would like to attempt to develop a unified framework that, despite clinical and diagnostic heterogeneities, provides a deeper understanding of the many facets of addiction.

Addictive disorders are characterized by (1) persistent and repetitive seeking toward specific targets, although negative consequences are expected, and (2) involvements of reward system activation in the brain. Existing models offer complementary accounts of addiction mechanisms, but no unified framework adequately captures the full complexity of these disorders. Although the mechanisms underlying addictions are believed to be similar, the different forms of addictions manifest in distinct phenotypic expressions (Alavi et al., 2012). Despite extensive research, addiction studies have yet to establish a standardized framework that comprehensively encompasses all forms of addictions. As so noted by West et al. (2019), “*The field of addiction struggles with a lack of clarity over many of its core constructs, with unresolved disputes over ways of representing and understanding the phenomena within its scope, and even debate over what falls within that scope*.” In a similar tone, Petry et al. (2018) argue “*Not only does the term “addiction” instigate controversy but so does consideration of new mental disorders.*”

As outlined above, the pronounced heterogeneity of addictive disorders highlights the need to clarify both shared and distinct features across SUD, BA, and FA. Are fundamental symptoms such as tolerance and withdrawal necessary and sufficient conditions in all types of addictions? What are possible relationships with other psychiatric disorders—which boundaries can be found between addictive disorders and other psychiatric disorders in which patients show addiction associated symptoms, such as obsessive compulsive disorder (OCD), impulse control disorder, and eating disorder?

The lack of consensus on a unifying model of addiction highlights the need for a conceptual framework that enables a better understanding of the full spectrum of addictive disorders. This article aims to propose a unified conceptual framework for the two recognized main subtypes of addiction—SUD and BA—while also considering the debated construct of FA. FA is conceptualized as an intermediate phenotype within the addiction spectrum, characterized by cue-triggered processes and pathogenic mechanisms that overlap with those observed in both SUD and BA (Vasiliu, 2021). In this framework, SUD, BA, and FA are understood as a spectrum of disorders, similar to the autism spectrum disorder, and are organized around the following unified key aspects: negative reinforcement, compulsive behaviors, cue-induced responses, and associative learning. These key aspects are considered shared main characteristics of the different addiction types, including SUD, BA, and FA.

## 2 Conceptualizing addictive disorders: cases of SUD, FA, and BA

Addictive disorders have been conceptualized within multiple theoretical frameworks (Clark, 2011), some of which are described in this section. Existing approaches to conceptualizing addictive disorders can be broadly grouped into two categories: (1) conceptualizing addictive disorders based on symptoms/clinical observations and (2) conceptualizing them based on neurobehavioral processes. The following paragraph will discuss the conceptualizations of SUD, BA, and FA.

### 2.1 Conceptualizations of SUD based on clinical approach

The definition of SUD is primarily based on observable clinical symptoms, which constitute the core characteristics of the disorder. According to the DSM-5, SUD is diagnosed based on 11 criteria, which are categorized into four overarching domains. Difficulties in one or more of these areas in relation to substance use continue to serve as the foundation for clinical diagnosis. These are loss of control over consumption (such as substance consumption in larger quantities or over a longer period than intended, repeated unsuccessful attempts to reduce or control consumption, significant time investment in acquiring, using, or recovering from substance use, and intense craving for the substance), social impairment due to substance use (such as neglect of work, school, or familial responsibilities, continued substance use despite recurring social or interpersonal conflicts, and abandonment or reduction of important activities in favor of substance use), risky or hazardous use (such as repeated substance use in situations that pose a danger and continued use despite awareness of associated physical or psychological harm), and physiological dependence (such as tolerance development, requiring higher doses to achieve the same effect and withdrawal symptoms upon cessation or reduction of substance use).

A similar diagnostic framework is employed in ICD-10, which classifies SUD under F1x.2—Dependence Syndrome. According to this classification, a diagnosis requires the presence of at least three of the following six criteria: i.e., strong desire or compulsion to use the substance (craving), impaired control over the initiation, amount, or cessation of use, tolerance development, necessitating increasing doses to achieve the same effect, withdrawal symptoms following reduction or discontinuation of substance use, neglect of alternative interests and activities due to substance use, and persistent substance use despite knowledge of its harmful physical, psychological, or social consequences, within a 12-month period.

While both the DSM-5 and ICD-10 emphasize observable behavioral patterns and physiological markers, the ICD-10 maintains a stricter distinction between harmful use (F1x.1) and dependence syndrome (F1x.2), whereas the DSM-5 integrates these concepts under a single diagnostic spectrum.

### 2.2 Conceptualizations of BA based on clinical approach

Similar to FA and in contrast to SUD, BA is not a separate category in the diagnostic catalogs, but rather a residual category. In DSM-5, gambling disorder (312.31 (F63.0)) can be found under nonsubstance-related disorders and is defined as follows: persistent and recurrent problematic gambling behavior leading to clinically significant impairment or distress. The clinical criteria listed below include increasing gambling for sustained arousal, irritability when trying to reduce gambling, loss of control over gambling behavior, excessive thoughts about gambling (e.g., plans or flashbacks), gambling as a coping mechanism for stress (e.g., negative feelings), and social and financial problems, such as lying, relationship problems or compensating for losses (“chasing losses”). There are also the number of other behavioral addictions that have not yet been recognized by the DSM-5 but included in ICD-11 (World Health Organization, 2018), such as Internet gaming disorder.

Several further models conceptualize addiction through clinical observations and characteristics. One prominent example is the Components Model of Addiction by Griffiths (2005), which lays a special focus on BA and defines behavior as addictive if it shares symptomatic overlaps with SUD in six specific components: salience, mood modification, tolerance, withdrawal, conflict, and relapse. Furthermore, this model suggests a three-step approach, which is identifying target behaviors as potentially addictive, screening them using the six inclusion criteria, and evaluating the biological and psychosocial risk factors associated with BA. Another example of conceptualization of BA based on clinical symptoms is the exclusion criteria model by Kardefelt-Winther et al. (2017). This model proposes that behavior should not be classified as BA if it meets the four criteria, such as that no other disorder can better explanation for symptom, the behavior is a willful choice, although potentially harmful and causes impairment, the behavior is persistence but causes no functional impairment or distress, and that the behavior is the result of a coping strategy.

Conceptualizing addiction based on symptoms and clinical observations is an extremely useful approach. However, the enormous heterogeneity of symptoms also clearly argues against such an approach. Most theories propose a defined framework that primarily implies one type of addiction, which also segregates the disorders in the intervention domain, which can lead to reduced treatment effectiveness (Kim and Hodgins, 2018). Another line of attempts to conceptualize addictions for BA cases is based on neurobehavioral processes, provided primarily by the idea that addiction is a disorder of associative learning (Robbins and Clark, 2015; Morris and Voon, 2016; James and Tunney, 2017; Perales et al., 2020). Similar theories have recently been put forward for SUD and FA. Etiological theories of SUD conceptualize the disorder as fundamentally behavioral, emphasizing learning processes and their behavioral consequences (Greener and Storr, 2023). In the following sections, this will be detailed, along with a rationale for why this approach would be useful in conceptualizing addictive disorders and how this approach fits into a dimensional model with clinical relevance.

### 2.3 Conceptualizations of FA based on clinical approach

FA is described as a behavioral manifestation in which highly palatable and therefore rewarding foods are consumed in quantities that exceed normal energy requirements (Kalon et al., 2016). As noted above, FA is not a formally recognized diagnosis in either ICD-10 or DSM-5. Accordingly, here the term FA is used in reference to the ongoing scientific debate, where it serves as a phenomenological construct describing addictive-like eating behaviors that show symptomatic overlaps with more well-defined addiction subtypes, such as SUD and BA (Vasiliu, 2021). Although the pathogenesis remains unclear, there appears to be substantial overlaps with SUD, particularly with respect to reward dysfunction, craving, and impulsivity (Vasiliu, 2021). Among other problems, FA is associated with a loss of control in the context of eating, which represents both psychological and physical stress for those affected (Fletcher and Kenny, 2018). “Hedonic hunger,” the concept referring to the phenomenon that food is consumed solely for the rewarding and good feeling, despite the absence of a physiological feeling of hunger (Espel-Huynh et al., 2018), is therefore at the heart of the disorder (Witt and Lowe, 2014).

In one perspective, FA may be considered more relevant to SUD. In their theoretical framework for defining FA, Kalon et al. (2016) use Sussman and Sussman’s five addiction criteria of SUD (Sussman and Sussman, 2011), which include participation in behaviors due to appetitive effects, significant expenditure of time thinking about, obtaining, using, and recovering from the effects of the behavior, satiation, described as a period of time immediately following use in which the addictive behavior temporarily subsides but quickly returns once the effects fade, impulsivity or loss of control over the behavior, and negative psychosocial, emotional, and health consequences.

Another set of studies suggests that FA may also be considered more relevant to maladaptive behavioral patterns, such as BA. From a clinical perspective, binge eating disorder (BED), a psychiatric condition understood as pathological overeating and the associated loss of control, is also suggested to fall under the umbrella term FA (Parylak et al., 2011). Bulimia nervosa (BN) is also a psychiatric condition characterized by pathological overeating followed by compulsive countermeasures such as vomiting or excessive exercise and thereby could meet the criteria of FA (De Vries and Meule, 2016). Overeating is seen as a possible symptom of FA and does not in itself represent a subcategory of FA (Fletcher and Kenny, 2018). For this reason, BED and BN have been implied with the term FA.

A clinical scale has been developed to quantify the severity of addictive-like eating, namely the Yale Food Addiction Scale (YFAS; Gearhardt et al., 2009), which was constructed to parallel DSM-IV criteria for substance dependence. Two validated versions of this instrument are currently available: the modified YFAS 2.0 (Schulte and Gearhardt, 2017), and the child version (dYFAS-C; Schiestl and Gearhardt, 2018), allowing the assessment of FA severity across adult and pediatric populations. However, despite the availability of these psychometric tools, there is currently no unanimously accepted and well-defined diagnostic criterion for food addiction in the literature (Vasiliu, 2021).

## 3 Reconstructing the concepts of psychiatric disorders with the dimensional model

In the conventional diagnostic system, psychiatric disorders are categorized with their symptoms. Although this system is useful in clinical practice, researchers have begun to be aware of its major limitations in the realm of psychiatric research. First, a profile of symptoms even within the same category of psychiatric disorder is highly heterogeneous in individuals. In addition, patients with one psychiatric disorder quite often tend to develop other psychiatric disorders, with the estimated prevalence of such co-morbidities of two disorders reaching up to 66% of all patients (Caspi and Moffitt, 2018). Although the existence of such critical problems has long been recognized, this problem has been neglected until quite recently. However, as basic research on psychiatric disorders has progressed and grown, it turns out now that many genes, molecules, and brain regions and networks are substantially overlapping with different classes of psychiatric disorder, and thereby it is not possible to clearly distinguish one disorder from another with such neuroscientific findings anymore.

Accordingly, these issues led researchers to reconceptualize psychiatric disorders from the currently adopted categorical model to dimensional model. In the categorical model, there is a (single) defect (which could be on a gene, neurotransmitter, brain region, etc.) that causes an assortment of symptoms, resulting in a specific category of psychiatric disorder, which is similar to, for instance, an infectious disease in which infection to viruses or bacteria causes an assortment of symptoms, such as fever, diarrhea, and so on. In the dimensional model, psychiatric disorder is considered a concept consisting of a bundle of neurobehavioral processes that are deviated from the average of the population, so that no single cause exists for symptoms of disorders.

A landmark in such reconceptualization of psychiatric disorder into the dimensional model is Research Domaine Criteria (RDoC), which is a project led by National Institute of Mental Health in the USA. RDoC is the framework consisting of six domains of human psychological/biological functions (Negative valence; Positive valence; Cognitive; Arousal/Regulatory; Sensorimotor; Social processes) (Insel et al., 2010). Each domain comprises constructs, which are neurobehavioral processes ranging from functional to dysfunctional. Constructs can be quantitatively measured using the method termed units of analysis, such as behavioral, physiological, and self-report data, for comprehensive understanding of the constructs.

## 4 Insights on addictive disorders from the dimensional model

### 4.1 Conceptualizing psychiatric disorders with neurobehavioral processes

The impacts of the dimensional model on our understanding of psychiatric disorder are particularly important in the following two points. First, psychiatric disorders in different categories of conventional nosology are continuous (= spectrum) and cannot be delineated. However, the idea of “spectrum” has already been incorporated into the diagnostic manuals at a certain extent; for instances, autism spectrum disorders (ASD) and schizophrenia spectrum disorders (SSD) that involve psychosis, impaired cognitive processes, unusual or disorganized behaviors affecting social activity, so that schizophrenia and relevant disorders, such as delusional disorder, schizoaffective disorder, and schizophreniform disorder, could be considered as a spectrum with different severity in specific aspects of symptoms (Ross and Margolis, 2019). Second, given that constructs are quantitative and continuously varied from functional to dysfunctional level, no clear delineation is possible between psychiatric and normal conditions. Thus, in the dimensional model, subjects with psychiatric conditions are considered as those residing in the extreme ends of normally distributed population (Sanchez-Roige and Palmer, 2020).

The notion of spectrum between healthy and pathological conditions in the dimensional model has already been suggested in SUD. Volkow et al. (2016) have argued that “*… as is the case for any other medical condition, there is a severity dimension … and that only a small percentage of those with a substance use disorder fall in the most severe category… this severity dimension and the mechanisms underlying the transition from mild to severe addiction …*” Accordingly, Volkow et al. (2016) have defined the word SUD as a diagnostic term for recurrent substance use that causes significant impairments, with its severity varying from mild to severe, whereas the term addiction refers to indicate only the most severe, chronic stage of SUD characterized by compulsive seeking of substances.

In contrast to SUD, quite some arguments have been made to go against this direction of the notion in BA. For instance, Flayelle et al. (2022) have stated “*…understanding and treating non–substance-related addictive behaviors rest on… (1) elucidation of the specific phenomenological characteristics of the emerging and possible behavioral addictions through priority recourse to qualitative research conducted in highly engaged individuals; (2) better delineation of high but healthy engagement (i.e., passion) and pathological involvement (i.e., disorder); and (3) endorsement of an approach that is not merely symptom or syndrome based, but is rather process based, thus reflecting the complexity of psychological functioning…*.” It is partly understandable why such statement has been made, as recent expansion of conditions that are referred to as BA (e.g., smartphone, exercise, work, etc.) appears as if almost any kind of (enthusiastic) behavior could be considered as addiction in the current situation. Nonetheless, it is unlikely that a clear delineation can be found that distinguishes behavioral addiction and highly engaged but non-pathological activities, such that behavioral addiction research should be directed to understand how addictive behaviors can be defined in the spectrum from healthy to pathological conditions rather than finding the delineation that distinguishes between them.

In the context of FA, this perspective supports the notion that the intensity and frequency of food consumption may gradually shift from necessary and healthy behavior or hedonic enjoyment to maladaptive patterns characterized by loss of control, particularly in response to highly palatable foods (Davis, 2014). Importantly, not only do specific types of foods function as potential cue-driven triggers, in a manner comparable to substances in SUD (McCausland et al., 2025), but there are also clear gradations in the severity of the associated pathological behaviors, such as craving and impaired control (Pelchat, 2002). Together, these observations indicate that FA can be understood as a condition that exists along its own continuum of severity and as an intermediate phenotype that integrates substance-like cue reactivity with behavior-driven compulsive patterns, thereby occupying a position between SUD and BA within the addiction spectrum.

### 4.2 Conceptualizing addictive disorders with neurobehavioral processes

The international Delphi consensus study has identified 7 neurobehavioral processes (constructs in RDoC) that are critical in explaining SUD (Yucel et al., 2019). These include 5 constructs from the positive valence domain (reward valuation, expectancy, action selection, reward learning, habit), one construct from the cognitive control domain (response selection/inhibition), and one more expert-initiated construct (compulsivity). If SUD, BA, and FA consist of a spectrum of the disorders, these neurobehavioral processes should mutually be involved in all of SUD, BA, and FA, and its conceptualizations should be based on these neurobehavioral processes.

Converging evidence indicates that SUD involves maladaptive associative learning (Di Chiara, 1999; Everitt and Robbins, 2005; Berridge and Robinson, 2016). Such associative learning in SUD includes not only operant conditioning, but also Pavlovian conditioning. Thus, substance seeking behavior (response) is associated with a substance as a reward (outcome); moreover, a process that an environmental cue (stimulus) is associated with a substance as a reward also takes place in development into the addicted state of SUD, which in turn causes cue-induced craving and relapse to the addicted substances in SUD patients. This associative learning theory of addiction has now been extended into conceptualization of BA (Robbins and Clark, 2015; Morris and Voon, 2016; James and Tunney, 2017; Perales et al., 2020), along with empirical supports from studies demonstrating cue-induced craving in gambling disorder (Crockford et al., 2005; Miedl et al., 2014), Internet and online gaming disorder (Niu et al., 2016; Zhang et al., 2016; Wegmann et al., 2018), and pathological buying (Trotzke et al., 2014). We have also recently demonstrated that patients with kleptomania exhibit altered responses to environmental cues associated with their symptoms, although whether these responses are associated with craving or relapse has remained unknown (Asaoka et al., 2023).

Robbins et al. (2012) have suggested that impulsiveness and compulsion play roles in an assortment of psychiatric disorders, with impulsivity associated with mania, personality disorders, and attention deficit/hyperactivity disorder (ADHD), whereas compulsion is associated with SSD, ASD, and OCD. In contrast, presence of both impulsiveness and compulsion characterizes SUD and eating disorders, although impulsivity and compulsivity may be convoluted in different stages of addiction, with impulsivity significant at intimal stage, whereas compulsion harsh at late chronic, stages of addiction (Yucel et al., 2019). In particular, impulsiveness is thought to be a risk factor for addiction, such that it plays prominent role at beginning of addiction process, whereas compulsivity is suggested to be developed through associative learning, and therefore, play major role in later, chronic stage of addiction (Everitt and Robbins, 2005). However, there is also a study reporting that cue-induced craving augments impulsivity in problem gamblers (Miedl et al., 2014). Thus, further investigation for relationships between impulsivity and compulsivity in relation to associative learning in addiction is required.

As mentioned above, FA not only shares similarities to various addiction subtypes such as SUD and BA within the psychological dimension, but also on a behavioral level. Existing evidence indicates overlap between the clinical phenotypes of SUD and FA, in regard to cue-driven-behavior (drug vs. hyperpalatable food), stress-coping, impulsivity and operant conditioning (Kalon et al., 2016). Similar to SUD, the consumption of highly processed foods is accompanied by strong craving and is no longer perceived as rewarding, but rather as an avoidance strategy to negative bodily sensations in FA (Meule and Kubler, 2012). These and other findings support the assumption that FA is subject to the same negative reinforcement mechanisms as SUD (Parylak et al., 2011) and consequently also BA (Alavi et al., 2012).

Collectively, some neurobehavioral processes are critical in conceptualizing addiction, including associative learning, impulsiveness, and compulsion.

### 4.3 Conceptualizing addictive disorders from their neural mechanisms

Addiction typically begins with a conscious and voluntary pursuit of highly rewarding stimuli or intoxication. The primary focus is on stimuli eliciting a rapid and substantial release of dopamine, such as psychoactive substances, high-calorie food, or gambling winnings, which are initially sought through deliberate, goal-directed behavior. However, as addiction progresses, this goal-oriented behavior gradually transitions into compulsion, characterized by a loss of conscious control over reward-seeking behavior (Uhl et al., 2019).

The role of compulsion in the context of underlying learning mechanisms is examined in greater detail later (in Section 5.1). The transition from goal-directed, consciously controlled behavior to compulsive engagement—a hallmark of addiction—is associated with neural alterations shared across multiple addictive disorders, including SUD and BA (Figee et al., 2016; DiFeliceantonio et al., 2018).

Central to the development of addiction is the mesolimbic system, a dopaminergic network primarily comprising the ventral tegmental area (VTA) and the nucleus accumbens (NAc). The VTA projects dopaminergic signals to the forebrain, thereby regulating reward perception and motivation. Simultaneously, additional pathways extend to the hippocampus and amygdala, integrating reward-related stimuli with memory and emotional processing. Strong reward stimuli such as abused drugs lead to an excessively high release of dopamine release. The “incentive-sensitization theory of addiction” presented by Berridge and Robinson (2016) explains that addictive drugs influence dopamine transmission in the mesolimbic system. Repeated drug use leads to neuroadaptive changes in this system, making the brain increasingly sensitive to drugs and drug-associated stimuli. As a result, the desire for the drug (craving) becomes stronger and stronger, even if the pleasure of the drug itself or the negative effects of withdrawal are reduced. Excessive dopamine release in the reward system leads to an upregulation of inhibitory receptor molecules in the NAc and VTA, resulting in local hypoactivity. Consequently, rewarding stimuli must be significantly stronger to induce the same subjective feeling of pleasure. Simultaneously, hypoactivity is also observed in the medial prefrontal cortex (PFC) (Khayat and Yaka, 2024). Over the course of the disorder, a functional impairment of the PFC becomes evident, primarily due to persistent PFC hypoactivity. This dysfunction is associated with various addiction-related symptoms, including impaired response inhibition, aberrant salience attribution, craving, and the development of compulsive behavior as part of a learning process (Goldstein and Volkow, 2011). There is evidence that these neurobiological patterns and changes are the same or similar in the various types of addiction, suggesting the same etiological mechanism (Grant et al., 2006; Passeri et al., 2023).

The mutual neural alterations in patients with different forms of addictive disorders, e.g., BA and SUD, is another indication of a shared neurological etiology. Thus, the ventromedial PFC shows hypoactivity in both BA and SUD patients in response to addiction-associated (e.g., gambling associated or drug associated) cues. A similar response in the frontal regions can also be observed in FA subjects with BED and BN when confronted with food cues (Volkow and Baler, 2014). Recent studies provide evidence that both the intake of cocaine and high-calorie and thus rewarding food can lead to a reduction in dopaminergic activity in the NAc (Alonso-Caraballo et al., 2021). At the same time, the consumption of high-calorie food, like the intake of cocaine and heroin, leads to reduced activity and alteration of receptors in the VTA (Figlewicz et al., 2003). Studies have also shown reduced activity in the VTA and NAc in cases of BA such as gambling disorder (Garcia-Castro et al., 2023).

**Figure 1:**
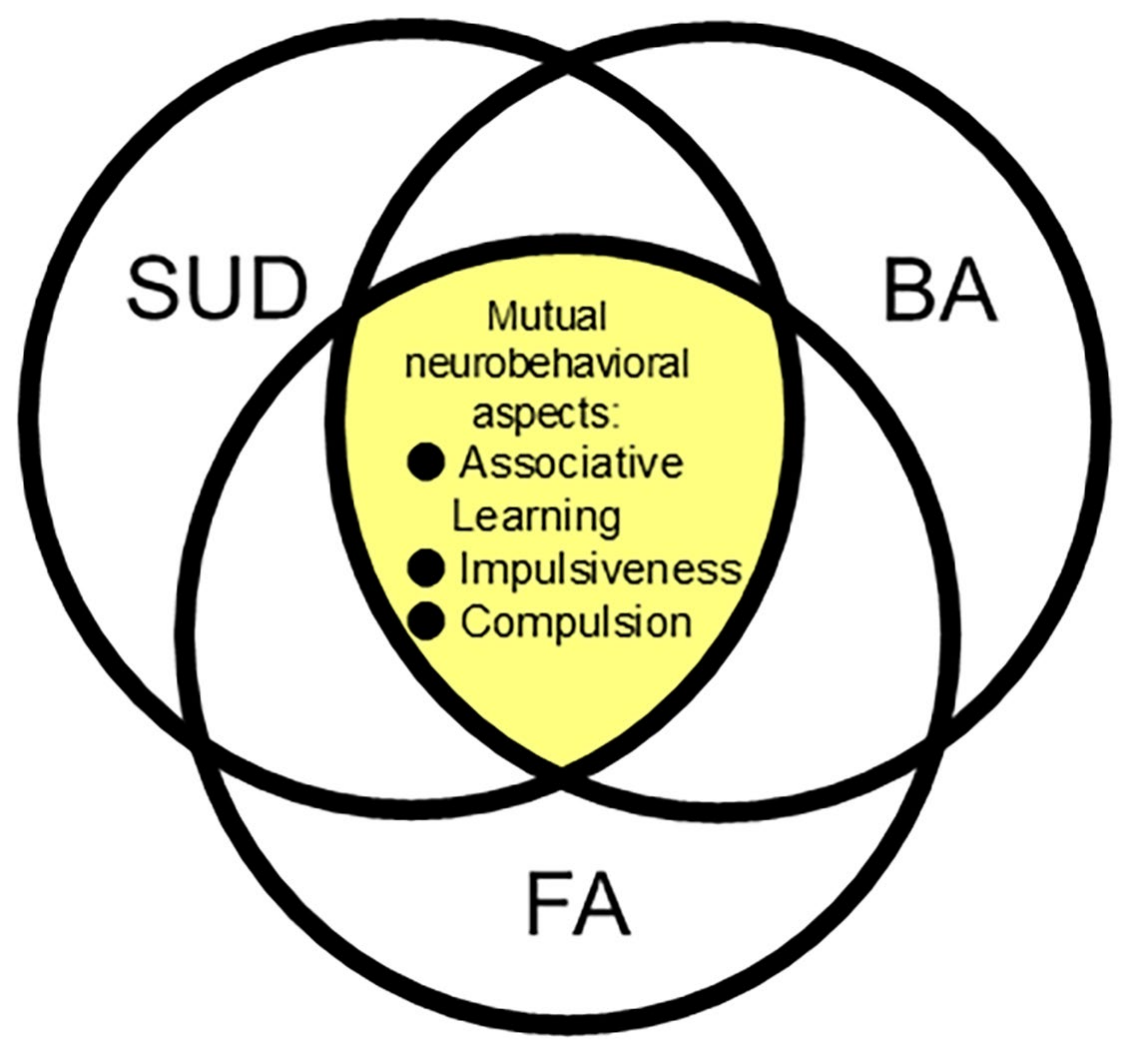
A schematic diagram illustrating mutual neurobehavioral mechanisms that underlie SUD (substance use disorder), BA (behavioral addiction), and FA (food addiction).

In summary, these studies described in this section show that different forms of addiction disorders, such as SUD, BA, and FA, share the same underlying neural mechanisms which lead to similar patterns in associative learning, impulsiveness and compulsion (Figure 1; Table 1).

**Table 1:** A summary of shared and distinct features of SUD, BA, and FA.

Dimension	Substance use disorders (SUD)	Food addiction (FA)	Behavioral addictions (BA)
Diagnostic status	Formally recognized diagnosis (DSM-5, ICD-10)	Not formally recognized; Debated construct	Partially recognized (e.g., Gambling disorder)
Primary trigger	Psychoactive substances	Highly palatable/ Ultra-processed foods	Behaviors (e.g., Gambling, Gaming)
Cue-induced craving	Cue-drug associations	Cue-food associations	Cue-behavior associations
Positive reinforcement (early stage)	Euphoria, Intoxication	Palatability, Hedonic eating	Excitement, Arousal
Negative reinforcement (later stage)	Avoidance of withdrawal, stress	Relief of craving, Negative affect	Relief of tension, Dysphoria
Loss of control/Compulsive seeking	Present	Present	Present
Associative learning	Pavlovian and instrumental conditioning	Pavlovian and instrumental conditioning	Pavlovian and instrumental conditioning
Impulsivity-compulsivity dynamics	Impulsivity (early) ➔ Compulsion (late)	Impulsivity (early) ➔ Compulsion (late)	Impulsivity (early) ➔ Compulsion (late)

## 5 Addictive disorders as a spectrum

The above arguments lead to the importance of how we could comprehensively understand the relationships between SUD, BA, and FA as “addiction” notwithstanding of their heterogeneities. By considering a conceptual framework of the dimensional model, we propose an idea that SUD, BA, and FA could be united as a spectrum of disorders. Here we argue three specific constructs imperative to compose such a spectrum. The first construct is that negative reinforcements rather than positive reinforcements could be a key to understanding addiction; the second construct is that cue-induced responses consequent of associative learning could be a unique characteristic to distinguish addiction from other resembling psychiatric disorders; and the third is that FA (which acts as its own spectrum, see the section 5.3) exhibits both aspects of SUD and BA, so that FA could be an intermediate phenotype between them in the spectrum. This spectrum is based on two neurobehavioral criteria that are inherent to all three types of addiction.

### 5.1 Negative reinforcement and compulsive seeking

Positive reinforcement with activation of the dopamine system is a neural basis and thereby the primary assertion of the current addiction research (Volkow et al., 2011; Wise and Robble, 2020). Thus, the presumption made for addictive disorders is that this positive reinforcement process is equally involved in any of SUD, BA, or FA.

A critical difference between SUD, BA, and FA, is that SUD as well as FA involve specific positive reinforcers that can over-stimulate the dopamine system beyond the physiologically ordinary level, although primary molecular targets are different between substances (Luscher and Ungless, 2006). On the other hand, although BA is also associated with positive reinforcements of actions as consequence of action execution, such reinforcement is dopamine activation within the ordinal level. Indeed, our behaviors are guided toward goals, which are reinforced at achievements of goals; however, majority of these actions do not get into addictive states.

Some aspects of environments, such as unpredictability may play a role in BA, for instance, such as gambling disorder, given that dopamine neuron firings have been shown to be associated with predictability of conditioned cues in reinforcement learning, with the strongest responses with a cue with 50% predictability (Fiorillo et al., 2003). However, such dopamine response is still a physiologically natural signal, and unlike exceedingly intense and sustained dopamine elevation with addictive substances. In addition, substance-induced dopamine release is significantly variable depending on substances, and therefore, the amount of dopamine release induced by the substances are clearly dissociated from being addicted to these substances (Nutt et al., 2015). These suggest that dopamine signals in positive reinforcement play a role not more than an association of an environmental cue and an outcome independent of whether the outcome is substances or not. Accordingly, positive reinforcement appears to be necessary for development of addictive states, but it is not sufficient and requires additional processes. Conceptualizing addiction from the commonality between SUD and BA is also difficult from the positive reinforcement perspective alone. This is partly attributable to the fact that positive reinforcers in BA are more heterogeneous and less clearly separable from negative reinforcement than those observed in SUD (e.g., stress or boredom reduction via mobile phone gaming versus the direct dopaminergic effects of alcohol consumption; Robinson and Berridge, 2008; Billieux et al., 2015).

In this regard, negative reinforcement appears to constitute a more central mechanism in both SUD and BA (Weatherly et al., 2012; Cho et al., 2019). One of the most consistent reports found in addiction research would be heightened negative affects, such as stress, anxiety, and depression, in patients with SUD (Koob and Le Moal, 1997; Sinha, 2008), BA (Blaszczynski and McConaghy, 1989; Matar Boumosleh and Jaalouk, 2017; Asaoka et al., 2020), and FA (Kayaoglu et al., 2023). Such negative affects may not necessarily be a consequence of withdrawals but could be a trigger (e.g., to alleviate daily stress). Indeed, they are demonstrated to play key roles in the initiation, maintenance, and relapse of SUD (Avery et al., 2016; Lemieux and al'Absi, 2016). Animal model studies have also demonstrated that rodents exposed to stress are more likely to self-administer addictive substances (Yap and Miczek, 2008).

The opponent process model of addiction by Solomon and Corbit (1978) also considers negative affects as the crucial aspect of addiction process, but along with positive ones. In this model, affective responses to stimuli consist of a fast and transient positive affective process (a-process) followed by a slow and prolonged negative affective process (b-process). Moreover, after repeated exposure to stimuli, although positive affects are not changed, the opponent negative processes are sensitized. Accordingly, substance taking in this model was driven initially by the net effect toward stronger positive processes, but in the later stage, i.e., addictive states, it becomes more negative affects predominance, and alleviation of negative affects drives substance taking. The allostasis model by Koob and Le Moal (1997) proposes a similar process with a three-stage cycle of binge/intoxication, withdrawal/negative affects, and preoccupation /anticipation, being addictive states after repeated substance intakes resulting in altered homeostatic state due to attenuated binge/intoxication (providing the basis for tolerance) and augmented withdrawal/negative affects.

These models thereby suggest that negative reinforcement is a prominent and crucial process, especially in the later stage of substance use, i.e., addictive states. This helps explain why not all reward-seeking, goal-directed behaviors develop into behavioral addictions and why individuals differ in addiction vulnerability despite comparable exposure to similar substances or behaviors.

However, positive and negative reinforcements closely interact with each other in the addiction process. Through the cyclical nature of addiction process, the balance of these two reinforcement processes may shift toward less significant in positive reinforcement due to tolerance and more prominent in negative reinforcement, respectively. In addition, while positive reinforcement is common for all the forms of addictive disorders, severity of negative reinforcement could vary depending on the type of disorder. For instance, negative reinforcement may play a more significant role in SUD that involves physical dependence than addictive disorders without physical dependence, including BA.

Negative reinforcement plays a central role in addiction and may be equally or more influential than positive reinforcement. First, negative reinforcement has been suggested to yield even stronger effects than positive reinforcement in associative learning; thus, unpleasant events tend to evoke relatively more attention, greater memory formation, and stronger and long-lasting changes in mood and emotion than positive events (Baumeister et al., 2001). Second, in the positive reinforcement paradigm, contingencies are arranged for operant response and delivery of the positive reinforcer at different timing, whereas in the negative reinforcement regimen, operant response reinforced by removal of punishment occurs under the presence of such punishment, so that, for instance, substance-seeking behavior in SUD and addicted behavioral process in BA could be more effectively reinforced to escape from negative affects. In this context, negative reinforcement provides a direct mechanistic link to compulsive seeking, here we primarily use this term to refer to cue-driven, repetitive motivational behaviors that precede and promote consummatory acts (taking), that is also a key feature of addictive disorders (Robbins et al., 2012; Yucel et al., 2019). The behavior is therefore no longer performed with a view to its rewarding consequences, but solely to escape negative consequences and emotional states. Thus, compulsive seeking can be understood as a behavioral manifestation of negative reinforcement processes in addictive disorders. This is particularly evident in OCD, where negative reinforcement is understood to drive the development and maintenance of compulsive behavior (Panny et al., 2024).

Collectively, in defining addiction as a spectrum, the role of negative reinforcement and compulsive seeking behaviors are highly useful constructs, as, unlike positive reinforcement that involves the problem of heterogeneity of rewards, it is a mutually crucial process between SUD, BA, and FA (Figure 1; Table 1).

### 5.2 Cue-induced responses in relation to associative learning

Given that mesolimbic dopamine system meditates reinforcement learning, the mechanism of addictive disorder has been suggested to involve maladaptive associative learning. Such learning theories of addiction incorporate the triangular relationship of Pavlovian conditioning, instrumental learning, and habit formation.

In the context of SUD, through Pavlovian conditioning, urges for substances (unconditioned responses elicited by outcomes, i.e., reinforcers) are associated with stimuli (situational cues, where substances are taken), such that conditioned situational cues consequently elicit urges for substances. Pavlovian conditioning could also play roles in BA; however, unlike SUD, reinforcers associated with conditioned stimuli are often indetermined in BA. For instance, reinforcers are complex and undecided in Internet, online gaming, and smartphone use (if these are really the forms of addictive disorders), whereas in others, such as gambling and kleptomania, initial reinforcers are relatively clear (e.g., money, merchandise).

Instrumental conditioning is an association of an action (response) with an outcome (reinforcer). In SUD, actions correspond to substance-seeking/taking behaviors, whereas in BA, actions vary depending on the types of BA, such as gambling, online gaming, and using Internet, for which, again, outcomes working as reinforcers are not well-defined. The instrumental response can be decreased by devaluation, i.e., response contingency degradation. This is somehow against the definition of addictive disorder, since in this disorder, uncontrolled behaviors are persistently and repeatedly delivered despite negative consequences. In addition, Pavlovian conditioning and instrumental learning are independent and could be confounding with each other.

Incentive sensitization (Berridge and Robinson, 2016) and incentive habits (Everitt and Robbins, 2005) models are two major associative learning theories that have been proposed in the mechanisms of addiction. The incentive sensitization model proposes that repeated experience of substance intakes overly amplifies “wanting,” along with sensitization of mesolimbic dopamine system, which induces inability to control aberrant motivation and devaluation-resistive, compulsive seeking of substances. Such sensitized drug-seeking and taking behaviors may involve Pavlovian-to-Instrumental Transfer, the mechanism that Pavlovian conditioned stimuli associated to a reinforcer could exert motivational influence to facilitate instrumental responding toward the reinforcer (Holmes et al., 2010). In contrast, the incentive habits model considers compulsive seeking and taking of substances as consequence of aberrant coupling of motivation to habits by stimulus–response (action) associations. Thus, instrumental responding to conditioned stimuli could be autonomous and resistant to extinction even if such instrumental responding is maladaptive and causes negative results.

Incentive sensitization model and incentive habits model are not mutually exclusive and could be complementary with each other. Studies have shown that a few times of exposures to addictive substances is sufficient to facilitate striatal dopamine release in healthy subjects, which is consistent with sensitization response to drugs. However, in chronic patients, decrease of dopamine release, along with down regulation of dopamine D2 receptors availability, in the striatum has been demonstrated (Nutt et al., 2015; Volkow et al., 2017). Such discrepancy could be explained by transition from sensitization to habit formation through progression of addictive state. In fact, not everyone who initially or recreationally taking drugs develop compulsive drug seeking and taking, and able to stop using drugs without treatments (Anthony et al., 1994). The transition from motivational to habitual responses is evident at both behavioral and neural levels. At the behavioral level, healthy human subjects exposed to only few times of psychostimulants, such as amphetamine, induces long-lasting sensitized striatal dopamine responses to the drugs and drug-associated environmental cues (Vezina and Leyton, 2009), suggesting that incentive sensitization is the mechanism that play roles even at relatively early stage of substance abuse. In contrast, functional imaging studies in SUD patients at a chronic stage of addiction found that striatal dopamine release is decreased along with down-regulation of dopamine D2 receptor availability, which could be the neural basis of tolerance (Nutt et al., 2015). In animal studies, drug-seeking responses could be reduced by devaluation early in training (Olmstead et al., 2001); however, such responses became insensitive to devaluation after extended training, suggesting that habitual drug seeking responses emerge in later, chronic stage of addiction (Zapata et al., 2010; Clemens et al., 2014). Consistent with these behavioral observations, neuroanatomical evidence supports a transition from motivational to habitual processes in addiction. Thus, neuroanatomical dopamine-dependent striato-nigro-striatal loop circuitry that connects the ventral to the dorsal striatum through recurrent connections with the midbrain dopamine system (Haber, 2014), enabling the intrastriatal shift from ventral striatum-dependent, motivational drug seeking and taking to dorsal striatum-dependent autonomous delivery of drug seeking and taking in response to environmental cues. In alcohol use disorder, a shift from motivational to habitual systems is reflected in decreased activation of neural substrates involved in goal-directed behavior and increased activation of circuits supporting habit formation (Vollstadt-Klein et al., 2010; Sjoerds et al., 2013).

A couple of insights can be gleaned from these models for conceptualizing a spectrum of addictive disorders. First, although substance seeking and taking are still goal-oriented in the incentive sensitization model, the incentive habit model suggests that substance seeking and taking are autonomous responses to stimuli and could be executed independent of outcomes. Thus, the incentive habit model is readily applicable to BA, in which unlike SUD, reinforcers such as substances are often indetermined. Second, it is of particular interest to note that both models explain the mechanisms of cue-induced craving and relapse in addictive disorders. Functional imaging studies have shown excessive striatal dopamine release in response to situational cue presentation in SUD patients (Volkow et al., 2011). A similar cue-induced dopamine release in the striatum is also observed in obese subjects, although it is unclear whether these subjects may strictly meet with the criteria of FA (Wang et al., 2001). Cue-induced craving has been demonstrated in BA, such as gambling disorder (Limbrick-Oldfield et al., 2017). Thus, cue-induced responses are the mutual constituent of addiction, and this process makes addiction distinguished from other psychiatric disorders. For instance, OCD involves compulsive thoughts and delivery of actions to alleviate negative affects, which are consistent with those of addictive disorders. However, OCD is strikingly different from addictive disorders in which compulsive behaviors in OCD are not cue-induced responses. OCD and addictive disorders are also different in pharmacotherapeutic responses, as naltrexone, which is the drug effective for treatments of SUD, including alcohol and opioid dependence (Singh and Saadabadi, 2025), as well as BA, such as gambling disorder (Ioannidis et al., 2025) and kleptomania (Grant and Kim, 2002), whereas naltrexone treatments even augment OCD symptoms (Amiaz et al., 2008). In contrast, antidepressants, which are effective for OCD treatments, yield little effect in addictive disorders (Torrens et al., 2005; Charney et al., 2015), collectively suggesting that the mechanisms that cause compulsions in OCD and addictive disorders could be different. These dissociated drug effects provide additional support for distinguishing OCD from addictive disorders. For instance, intermittent explosive disorder and kleptomania are categorized into OCD, but although kleptomania exhibits impulsive and compulsive aspects of behaviors, intermittent explosive disorder is characterized by impulsive execution of behaviors. Naltrexone has been shown to improve kleptomania symptoms, but not intermittent explosive disorder, whereas antidepressant treatments are effective in intermittent explosive disorder, but controversial or even worsening kleptomania symptoms (Grant, 2006; Tahir et al., 2022), suggesting that kleptomania, but not intermittent compulsive disorder, resembles more like addictive disorder. In accordance with this argument, Asaoka et al. (2023) have recently shown that kleptomania patients exhibit altered gazing and prefrontal cortical responses to the cues associated with their symptoms.

### 5.3 FA as an intermediate between SUD and BA

FA is conceptually both old and new (Meule, 2015), as the concept of FA had already been coined by Randolph (1956), but its research has barely been conducted thereafter up until recently, when its research has flourished since introduction of the assessment criteria for FA, which parallels the DSM-5 criteria of SUD, YFAS by Gearhardt et al. (2009). Although FA is not recognized as a psychiatric disorder in the current DSM-5, FA is characterized by symptoms resembling those of SUD and fulfill the criteria of addictive disorders, such as the loss of or reduced control over intake, experience of cravings, and intake of the amount more than expected (Gordon et al., 2018). The concept of FA is further supported by studies, such as a functional imaging study in subjects with obesity (Wang et al., 2001) and binge eating disorder (Wang et al., 2011), who are not necessarily met the criteria of FA, but demonstrate enhanced cue-induced striatal dopamine release. Nonetheless, there are still significant debates about the concept of FA and has still been challenged by the significant number of researchers (Finlayson, 2017; Fletcher and Kenny, 2018), and a recent study has reported that obesity has limited behavioral overlaps with addictive disorders and psychiatric phenotypes (Vainik et al., 2020).

Interest in FA may be partly explained by its conceptual proximity to BA, as BA does not involve addictive substances, and positive reinforcers in BA are not always clear with dopamine activation supposed to be at physiologically natural range. In FA, however, the positive reinforcers are foods. Specific types of foods are suggested to be important in the construct of FA. Thus, consumption of refined foods with high glycemic load (GL), i.e., foods rich with fats, sugars, sodium, and carbohydrates (e.g., pizza, chocolate, chips), are suggested to stimulate the reward system more than other natural foods (e.g., vegetables) (Schulte et al., 2015), such that positive reinforcements with these processed foods could be similar in the sense of substances in SUD. Indeed, this is similar, if not identical, to the fact that a coca leaf itself does not yield dependence (Weil, 1978), whereas when it is processed into cocaine, it demonstrates dependence. Thus, refined psychoactive substances become highly addictive, as they can be taken into the body substantially at once, resulting in strong stimulation of the reward system.

Another line of evidence suggests that FA exhibits characteristics that are consistent with eating disorders, particularly BED, BN, and anorexia nervosa (AN) with binge-purge subtype (Wiss and Avena, 2020). These eating disorders are characterized by recurrent episodes of binge eating in which abnormally large quantity of foods than intended is consumed within a short period of time, along with feelings of loss of or reduced control and consequent emotional distress. Subjects with BED are also suggested to overeat to decrease mental problems, such as depressive symptoms, rather than pursuing a specific food or taste (Leehr et al., 2015). These overlap substantially with those observed in addictive disorders, suggesting that FA could be a problem of eating behavior itself (behavioral process) rather than the specific food type to be ingested. This notion is supported by epidemiological surveys unveiling that prevalence of subjects who meet the criteria of FA is particularly high in subjects with eating disorders (Hebebrand et al., 2014). In addition, the prevalence is also high not only in obese subjects, but also in underweight people, which could be associated with AN with binge-purge subtype (Hauck et al., 2020). In the condition of severe food deprivation, individuals have also been shown to exhibit binge-like consumption for less refined carbohydrate and nutritionally low-fat foods (Keys et al., 1950).

Leading researchers in the field argue that these different perspectives, which suggest that FA resemble either more like SUD or BA, are incompatible with each other (Gearhardt and Hebebrand, 2021). However, although high GL foods play a role in FA, it appears that high GL foods are not required all the time in the process of being addictive. For instance, animal model studies have shown that although rodents usually do not binge-eat nutritionally standard chows, they over-consume these nutritionally standard chows when they are pre-exposed to high fat and sugar chows (Hagan et al., 2003). Moreover, when environmental cues are associated with high fat and sugar chows, cue presentations promote binge eating for nutritionally standard chows, i.e., cue-induced responses (Boggiano et al., 2009). Thus, once in the addictive state, refined foods are no longer important in compulsive food consumption, complying with the incentive habit model. Indeed, although YFAS, the assessment of FA, are based on SUD similarities, this assessment is clearly reliant on behavioral attributes (e.g., impaired control over food consumption), which suggest that the concept of FA posits an interaction of the specific type of foods that are disposed to be addicted (e.g., high-fat, high-sugar foods) and behavioral engagements (e.g., eating to cope with negative affects). Both aspects of SUD and BA in FA are also not separable in which individuals seeking specific substances within foods (e.g., fat, sugar) could develop a behavioral pattern over time to crave the quantity of foods (consumption more than expected), whereas individuals who originally seek the quantity of foods (i.e., BED, BN) rather than particular substances (e.g., fat, sugar) could eventually become dependent on these certain substances within foods even they were unknown to the substances (Bak-Sosnowska, 2017).

**Figure 2:**
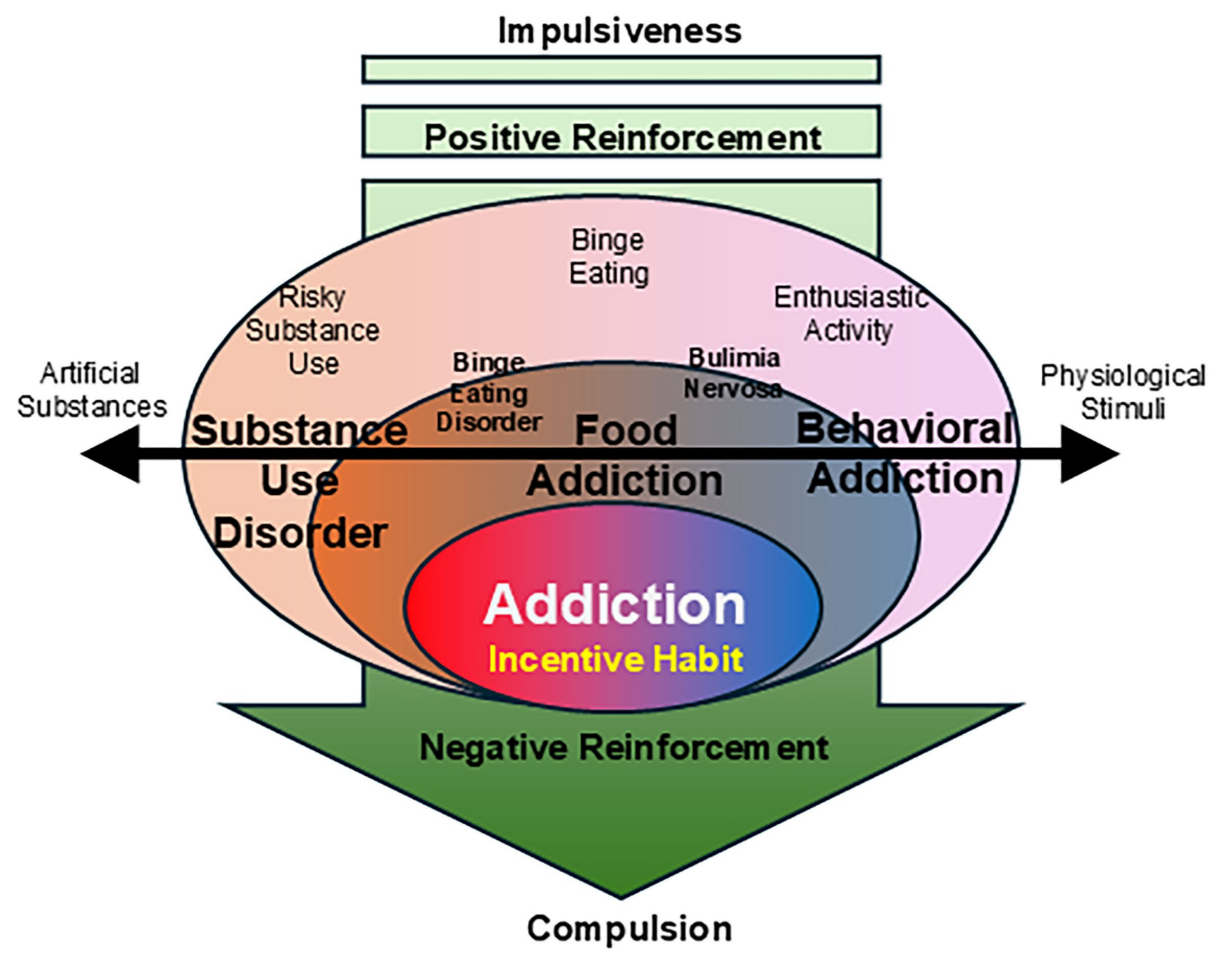
A schematic diagram illustrating the framework of addiction spectrum disorder.

Collectively, FA is not incompatible between SUD and BA but could be considered as a condition characterized by both aspects of SUD and BA, and thereby an intermediate phenotype between them, forming a spectrum with SUD, BA, and FA under the umbrella term of “addiction spectrum disorder” (Figure 2). Within this spectrum, FA could represent a kind of spectrum of its own, which in turn is defined by the two main representatives of the umbrella term, BED and BN. BED is closer to SUD, as the pathological consumption of highly rewarding foods and the associated loss of control is closer in concept to impulsive and, later on, compulsive drug use. BN, on the other hand, borders BA within the spectrum, as this addiction involves a stronger behavioral component characterized by compulsive countermeasures such as vomiting or excessive exercise (Figure 2).

Integrating FA into the addiction spectrum concept could be significant importance for clinical intervention. The current diagnostic manuals group various eating-associated eating-related behavioral phenotypes under the umbrella term “eating disorder.” This includes not only BED and BN but also AN. In contrast to SUD in which the mesolimbic system is often overactivated that increases dopamine release, there seems to be inherently a deficient connectivity within the mesolimbic system from the outset in AN (Tadayonnejad et al., 2022). This reduced connectivity causes insufficient dopamine release to potential reward stimuli, lowering motivation to pursue rewards. Thus, the neurobiological profile of AN appears to be distinct from addictive disorders but closer to that of OCD, which may not be surprising given the frequent comorbidity of both conditions (Levinson et al., 2019). Reevaluating the current classifications of addictive disorders, eating disorders, and other psychiatric disorders with impulsiveness and compulsion could, therefore, not only provide a more meaningful framework for future research but also help make therapies more disorder-specific and thus more efficient in clinical practice.

## 6 Addiction spectrum disorder as a model

Below, a model is presented that integrates various forms of addiction—including SUD, FA (specifically binge-eating disorder [BED] and bulimia nervosa [BN]), and BA—under the premise of a shared neurobehavioral etiology (Figure 2). This framework is based on two key mechanisms: (1) negative reinforcement and compulsive seeking, and (2) cue-induced responses in relation to associative learning.

At the onset, an above-average level of impulsivity may drive individuals to seek immediate reward stimuli, particularly in response to stress. These stimuli may take the form of risky substance use, binge eating, or engaging in highly stimulating activities, such as gambling, which provide a short-term experience of heightened pleasure. Central to all these behaviors is the immediate and consciously perceived sense of rewards.

Through processes of positive reinforcements, these stimuli become increasingly associated with stress relief and rewards, leading initially dysfunctional behaviors to develop into incentive-driven habits and, ultimately, into addictive patterns. The persistence of addiction is reinforced when the primary mechanism shifts from positive to negative reinforcements—such as when substances are consumed primarily to avoid withdrawal symptoms, or when an initially pleasurable activity is continued primarily to prevent the additional stress associated with its cessation. It is important to emphasize that this transition is not linear; rather, positive and negative reinforcement alternate in dynamic cycles and may vary across different stages of addiction. In early phases, hedonic and rewarding effects constitute the predominant form of positive reinforcement. As the disorder progresses, however, negative reinforcement — such as the alleviation of stress or dysphoric states — becomes increasingly dominant. Importantly, both reinforcement processes coexist at any given time, with their relative contribution depending on progressive neurobiological adaptations and imbalances within the nervous system (Solomon and Corbit, 1978; Koob and Le Moal, 2001). Through this mechanism, along with cue-induced learning, addiction-associated stimuli can elicit “craving” in affected individuals, ultimately transforming the initially conscious pursuit of rewarding stimuli into a compulsive behavior.

The spectrum of disorders that can be attributed to this fundamental mechanism ranges from SUD, characterized by the misuse of exogenous substances which are artificially designed to release an unnatural amount of dopamine in mesolimbic structures, and BA, which are driven by endogenous physiological stimuli such as winning experience resulting from a highly engaging non-substance-related behaviors. Notably, FA occupies an intermediate position within this continuum, incorporating elements of both SUD and BA. Specifically, BED aligns more closely with SUD due to its resemblance to substance misuse (in this case high-sugar food for instances), whereas BN shares greater similarities with BA, given its cyclical and compulsive behavioral patterns, where highly engaging non-substance-related behaviors such as excessive sport can lead to some sort of reward-related outcome expectancy. Therefore, FA not only serves as a bridge between SUD and BA—encompassing aspects of both depending on the specific type of FA considered—but also forms a continuum in itself, linking all addiction types, including their subtypes, into a broader addiction spectrum.

## 7 Practical implications

### 7.1 Addiction spectrum in diagnosis

A possible integration of the addiction spectrum into existing diagnostic systems could be based on the presentation of ASD or SSD in ICD-10. In these classifications, the cardinal symptoms of the respective spectrum are defined first. Depending on the number of symptoms fulfilled, the extent of the disorder is then divided into different levels of severity. This is followed by a detailed overview and a brief description of the respective clusters represented in the spectrum. A similar structure could also be used for the addiction spectrum disorder.

Compulsion and cue-induced craving could be placed in front as cardinal symptoms of the addiction spectrum disorder. The fulfillment of both symptoms would be a prerequisite for the diagnosis and for further diagnostics within the spectrum. Other symptoms could be based on the current DSM-5 criteria for SUD, such as loss of control, continued behavior despite social or health problems, desire to stop the behavior but no ability to do so, withdrawal symptoms, and that depending on the number of symptoms met, the addictive disorder could be assigned to a corresponding level of severity. As in ICD-10, the classification of addiction disorder could be divided into different clusters, each representing specific types of disorder as Cluster 1: SUD (substance-related addiction), Cluster 2: FA (both substance-related and behavior-related), and Cluster 3: BA (behavioral addictions). This categorization enables a differentiated and individualized diagnosis of addiction disorders and could provide a sound basis for clinical practice and the development of therapeutic approaches, such as all-encompassing screening procedures that capture the various addictions and addiction risks and can thus capture an individual and differentiated addiction profile of the patient. It is important to note that the proposed spectrum model does not include mutually exclusive criteria. Individuals can meet the criteria of multiple clusters or shift between clusters over time, reflecting comorbidity and so-called addiction transference. Within this framework, such patterns are conceptualized as shifts or overlaps within the same spectrum, driven by shared mechanisms such as stimulus reactivity, impulsivity, and dysfunctional regulation of negative affects.

### 7.2 Possible screening-approaches

By focusing on neurobiological and neurobehavioral profiles, such an addiction spectrum anchored in diagnostic frameworks would not only be able to incorporate psychiatric conditions that have presumably been addictive disorders but less studied to date (e.g., hypersexual disorder, internet gambling disorder, compulsive buying disorder), but it would also allow for comprehensive screening. This could facilitate the creation of a more individualized addiction profile and enable preventive monitoring. Today, the concept that various addiction types share similar neurological patterns and behavioral phenotypes is widely accepted in the scientific community (Schreiber et al., 2013; Passeri et al., 2023). For this reason, dual or more comprehensive addiction screening has been recommended. Meta-analyses indicate that individuals with FA are more susceptible to SUD (Bahji et al., 2019) Furthermore, there is familial clustering of addictions along the spectrum. For instance, studies have shown that SUD patients are more frequently found in the immediate family circles of individuals with pathological gambling (BA patients) (Black et al., 2014). Given a shared etiology with common core elements, both the familial prevalence and the significant comorbidities of the individual addiction clusters are not unexpected. It is therefore crucial that clinicians are sensitized to a comprehensive, holistic understanding of addictive disorders. This approach is not only essential for individualized and thorough interventions but also for optimal prevention. For instance, patients with FA may switch their addictive behaviors after certain interventions and develop a SUD (addiction transfer) (Chiappetta et al., 2020).

Defining addictive disorder as a spectrum allows for comprehensive screenings, which could be of significant relevance in everyday clinical practice. Such screenings address all addictive tendencies and risks, thus helping to prevent phenomena like addiction transfer. Differentiated prevention is achieved by practitioners working with a comprehensive addiction profile based on the spectrum concept, rather than focusing on just one subcategory during intervention. Such a screening should comprehensively capture the patient’s individual addiction history, along with any relevant risk factors. Additionally, the diagnostic process should prioritize the assessment of neurobiological and neurobehavioral indicators. In this context, certain spectrum-specific behavioral phenotypes—such as compulsivity, cue-induced craving, impulsivity, and risk-taking tendencies—could serve as critical screening criteria. This approach would not only account for the general predisposition to addiction but also facilitate the identification of distinct addiction types and their varying degrees of severity, thus positioning the individual within the broader addiction spectrum. This could be accomplished through the combined use of standardized questionnaires, diagnostic interviews, or cognitive tests, such as the Iowa gambling task (Bechara et al., 1994) or the Stroop test for impulse control (Stroop, 1935), which provide valuable insights into control processes and impulsiveness. Furthermore, cue-induced craving and other underlying neurobehavioral phenomena could be assessed using tasks like stop-and-go tests, which are designed to measure response inhibition and behavioral control.

### 7.3 Research and social implications

Similar to screening diagnostics, research could employ methodologies that facilitate conclusions regarding a shared neurobehavioral and neurobiological etiology inherent to the addiction spectrum. To empirically substantiate the premise of an addiction spectrum, a range of experimental designs should be utilized to ensure the most comprehensive investigation of the theoretical framework.

Experimental paradigms involving cohorts representing various addiction subtypes within the spectrum, alongside corresponding control groups, could provide insights into shared underlying mechanisms. For instance, by comparing the performance of different addiction groups, as well as subgroup variations within the experimental cohort with cognitive tasks such as the Iowa Gambling Task and the Stroop Test employed to assess both general impulse control and compulsivity, meaningful patterns may emerge. The Iowa Gambling Task has consistently demonstrated impaired decision-making and dysfunctional executive regulation across multiple addiction types (Kovacs et al., 2017). Given that executive control deficits influence not only impulsivity but also compulsivity, such findings could further support the conceptualization of addiction as a spectrum disorder (Kurt et al., 2017).

Cue-exposure paradigms can be employed to experimentally investigate the second primary psychological mechanism underlying addictive disorder: cue-associated craving and learning. Additionally, a modified Implicit Association Test (IAT) could provide valuable insights into implicit attitudes and associations that may be difficult or impossible to capture through self-report questionnaires due to social desirability bias. The IAT evaluates reaction times between paired concepts with varying degrees of implicit association (Greenwald et al., 1998), such as alcohol and positive (incongruent condition) versus alcohol and negative (congruent condition). In addiction studies, modified versions of the IAT have been developed to assess different types of addictive behaviors (Lindgren et al., 2013; Roh et al., 2018). Furthermore, neuroimaging data can be collected during the IAT to examine potential differences in neural activation patterns between experimental and control groups. This approach enables the identification of intensity variations in brain activity associated with addiction-related cognitive processing. For instance, research suggests that compulsivity may be linked to altered EEG patterns (Perera et al., 2023), highlighting the potential for neurophysiological markers to further elucidate the underlying mechanisms of addictive behaviors.

To further understand the neurobiological processes inherent to the addiction spectrum, it would also be feasible to employ targeted knock-out animal models of the relevant genes to observe how they respond to addiction-associated cues, such as substances like cocaine, high-glucogenic food, or gambling-like tasks, including variable reward paradigms. Moreover, toxic lesions in animal models could be utilized to investigate the influence of ventromedial PFC dysfunction more thoroughly by means of targeted kainic acid injections, which simulate addiction-specific hypofrontality. This approach allows for the observation of how the model develops a specific type of addictive behavior when confronted with corresponding stimuli.

Furthermore, twin studies provide evidence of genetic family clustering of different types of addiction. For example, patients with BA often have first-degree SUD relatives (Grant et al., 2006). The correlation between addictive disorders and specific genetic polymorphisms remains incompletely understood. However, there is some evidence suggesting that both addiction-specific and general addiction-related genetic variations are involved, such as those in monoamine oxidase A (MAOA), the serotonin transporter (SLC6A4), and catechol-O-methyltransferase (COMT) (Ducci and Goldman, 2012). Meta-analyses have identified approximately 19 independent single-nucleotide polymorphisms that are significant for the general mechanism of addiction (Hatoum et al., 2023).

## 8 Limitations

Several major limitations should be acknowledged in the present proposed model.

First, it should be noted that, as discussed above, FA is not currently recognized as a distinct diagnostic entity in either the DSM-5 or the ICD-10. Accordingly, the addiction phenotype referred to here as “food addiction” should be understood as a phenomenological and research-oriented construct rather than an established clinical diagnosis, and it requires further systematic investigation.

Second, the present framework is derived from conceptual integration of existing literature and does not introduce new empirical data. In particular, the neurobiological arguments supporting an addiction spectrum are largely informed by findings from animal models, which raises important questions regarding their direct applicability to human addictive behaviors.

Consequently, future research employing empirical and clinical approaches will be necessary to systematically test and validate the proposed addiction spectrum in human populations and to substantiate the theoretical assumptions outlined in this review.

Furthermore, the model we propose is particularly relevant for psychotherapeutic interventions, as it addresses transdiagnostic mechanisms such as cue reactivity, craving, and compulsive seeking. Pharmacological treatment approaches, in contrast, remain largely tied to specific diagnoses and clinical manifestations. Accordingly, the present model is not intended to replace nosology-based pharmacotherapy, but rather to complement it by informing the development of psychotherapeutic strategies.

Finally, within a dimensional addiction spectrum, comorbidity and so-called “addiction transfer” can be understood as shifts along shared neurobehavioral dimensions rather than as the emergence of independent disorders. From this perspective, fundamental mechanisms such as negative reinforcement, cue-induced craving, and compulsive seeking persist, while the specific addictive target may change depending on environmental factors, availability, and individual vulnerability. Comorbidity therefore represents an expected feature of a spectrum-based model of addiction rather than an exception.

## 9 Conclusion

In this article, we have proposed three issues specifically to be considered in conceptualizing addictive disorders that unite SUD, BA, and FA as a spectrum of disorders. The fact that all forms of addictive disorders inherent similar neurobehavioral mechanisms serve as a common premise for an addiction spectrum model.

In particular, we have first proposed focusing on negative reinforcements, instead of positive reinforcement aspect that has traditionally been considered in the mechanisms of addictive disorders but substantially heterogeneous between SUD, BA, and FA as well as within each of these disorders. Such negative reinforcements are associated with compulsivity, and therefore, the neural mechanisms of compulsion in relation to negative reinforcements could provide important insights on an addictive disorder conceptualization. Second, cue-induced responses in relation to associative learning, in particular stimulus–response associations in habit formation, may be useful in explaining the mutual mechanisms for SUD, FA, and BA, and could be a unique characteristic that distinguishes addictive disorders from other psychiatric disorders, such as OCD in which compulsion is a major behavioral problem. Then, we have focused on the current debate of FA in the field. Instead of arguing whether FA is considered as either SUD or BA, we have proposed that this specific addictive condition may be more suitable to locate in the middle of SUD and BA, such that FA is an intermediate phenotype exhibiting both aspects of SUD and BA, filling the gap between them and forms its own spectrum where BED is closer related with SUD and BN with BA, respectively.

Despite increasing de-stigmatization of individuals with mental illnesses and widespread awareness campaigns, SUD is still largely regarded as primarily moral issues (Frank and Nagel, 2017). People affected by these disorders experience less understanding and more rejection in daily life. For instance, only 22% of respondents would voluntarily collaborate at work with someone known to have drug problems, whereas 62% of respondents would be willing to work with someone who has mental health issues (Barry et al., 2014). This discrepancy and public rejection ultimately lead to a breakdown in social networks, resulting in loneliness, which, in turn, is one of the major risk factors for SUD (Hosseinbor et al., 2014). To break this vicious cycle, a unified understanding of addictive disorder must be established, one that clearly demonstrates that this phenomenon is not a moral failing of the individuals affected, but a disease that can be understood neurobiologically and neurobehaviorally, just like any other medical condition. Framing addictive disorder within a spectrum could help de-stigmatize the term “addiction,” particularly in relation to SUD. Studies, for example, show that societal attitudes toward people with eating disorders are significantly more positive (Mond et al., 2006). This perspective suggests that addiction-like behaviors are more readily recognized as pathological rather than moralized, which may facilitate more supportive social responses. It can therefore be assumed that, for example, SUD could be de-stigmatized if it is argued that it shares similar etiological factors with eating disorders such as BN and BED. This shift in societal attitudes could provide affected individuals with more social support and stability.

## Data Availability

The original contributions presented in the study are included in the article/supplementary material, further inquiries can be directed to the corresponding author.

## References

[ref2] Alavi S. S. Ferdosi M. Jannatifard F. Eslami M. Alaghemandan H. Setare M. (2012). Behavioral addiction versus substance addiction: correspondence of psychiatric and psychological views. Int. J. Prev. Med. 3, 290–294. 22624087 PMC3354400

[ref3] Alonso-Caraballo Y. Fetterly T. L. Jorgensen E. T. Nieto A. M. Brown T. E. Ferrario C. R. (2021). Sex specific effects of "junk-food" diet on calcium permeable AMPA receptors and silent synapses in the nucleus accumbens core. Neuropsychopharmacology 46, 569–578. doi: 10.1038/s41386-020-0781-1 32731252 PMC8027187

[ref4] American Psychiatric Association (2013). Diagnostic and Statistical Manual of Mental Disorders. 5th Edn. Washington: American Psychiatric Association.

[ref5] Amiaz R. Fostick L. Gershon A. Zohar J. (2008). Naltrexone augmentation in OCD: a double-blind placebo-controlled cross-over study. Eur. Neuropsychopharmacol. 18, 455–461. doi: 10.1016/j.euroneuro.2008.01.006 18353618

[ref6] Anthony J. C. Warner L. A. Kessler R. C. (1994). Comparative epidemiology of dependence on tobacco, alcohol, controlled substances, and inhalants: basic findings from the National Comorbidity Survey. Exp. Clin. Psychopharmacol. 2, 244–268. doi: 10.1037/1064-1297.2.3.244

[ref8] Asaoka Y. Won M. Morita T. Ishikawa E. Goto Y. (2020). Heightened negative affects associated with neurotic personality in behavioral addiction. Front. Psych. 11:561713. doi: 10.3389/fpsyt.2020.561713 PMC7495191 33101082

[ref9] Asaoka Y. Won M. Morita T. Ishikawa E. Goto Y. (2023). Distinct situational Cue processing in individuals with kleptomania: a preliminary study. Int. J. Neuropsychopharmacol. 26, 340–349. doi: 10.1093/ijnp/pyad005 36728203 PMC10229848

[ref10] Avery S. N. Clauss J. A. Blackford J. U. (2016). The human BNST: functional role in anxiety and addiction. Neuropsychopharmacology 41, 126–141. doi: 10.1038/npp.2015.185 26105138 PMC4677124

[ref11] Bahji A. Mazhar M. N. Hudson C. C. Nadkarni P. Macneil B. A. Hawken E. (2019). Prevalence of substance use disorder comorbidity among individuals with eating disorders: a systematic review and meta-analysis. Psychiatry Res. 273, 58–66. doi: 10.1016/j.psychres.2019.01.007 30640052

[ref12] Bak-Sosnowska M. (2017). Differential criteria for binge eating disorder and food addiction in the context of causes and treatment of obesity. Psychiatr. Pol. 51, 247–259. doi: 10.12740/PP/OnlineFirst/62824 28581535

[ref13] Barry C. L. Mcginty E. E. Pescosolido B. A. Goldman H. H. (2014). Stigma, discrimination, treatment effectiveness, and policy: public views about drug addiction and mental illness. Psychiatr. Serv. 65, 1269–1272. doi: 10.1176/appi.ps.201400140 25270497 PMC4285770

[ref14] Baumeister R. F. Bratslavsky E. Finkenauer C. Vohs K. D. (2001). Bad is stronger than good. Rev. Gen. Psychol. 5, 323–370. doi: 10.1037/1089-2680.5.4.323

[ref15] Bechara A. Damasio A. R. Damasio H. Anderson S. W. (1994). Insensitivity to future consequences following damage to human prefrontal cortex. Cognition 50, 7–15. doi: 10.1016/0010-0277(94)90018-3 8039375

[ref16] Berridge K. C. Robinson T. E. (2016). Liking, wanting, and the incentive-sensitization theory of addiction. Am. Psychol. 71, 670–679. doi: 10.1037/amp0000059 27977239 PMC5171207

[ref17] Billieux J. Maurage P. Lopez-Fernandez O. Kuss D. J. Griffiths M. D. (2015). Can disordered mobile phone use be considered a behavioral addiction? An update on current evidence and a comprehensive model for future research. Curr. Addict. Rep. 2, 156–162.

[ref19] Black D. W. Coryell W. H. Crowe R. R. Mccormick B. Shaw M. C. Allen J. (2014). A direct, controlled, blind family study of DSM-IV pathological gambling. J. Clin. Psychiatry 75, 215–221. doi: 10.4088/JCP.13m08566 24500179 PMC4221079

[ref20] Blaszczynski A. Mcconaghy N. (1989). Anxiety and/or depression in the pathogenesis of addictive gambling. Int. J. Addict. 24, 337–350. doi: 10.3109/10826088909047292 2793286

[ref21] Boggiano M. M. Dorsey J. R. Thomas J. M. Murdaugh D. L. (2009). The Pavlovian power of palatable food: lessons for weight-loss adherence from a new rodent model of cue-induced overeating. Int. J. Obes. 33, 693–701. doi: 10.1038/ijo.2009.57 PMC2697275 19350040

[ref23] Caspi A. Moffitt T. E. (2018). All for one and one for all: mental disorders in one dimension. Am. J. Psychiatry 175, 831–844. doi: 10.1176/appi.ajp.2018.17121383 29621902 PMC6120790

[ref24] Charney D. A. Heath L. M. Zikos E. Palacios-Boix J. Gill K. J. (2015). Poorer drinking outcomes with citalopram treatment for alcohol dependence: a randomized, double-blind, placebo-controlled trial. Alcohol. Clin. Exp. Res. 39, 1756–1765. doi: 10.1111/acer.12802 26208048

[ref25] Chiappetta S. Stier C. Hadid M. A. Malo N. Theodoridou S. Weiner R. . (2020). Remission of food addiction does not induce cross-addiction after sleeve gastrectomy and gastric bypass: a prospective cohort study. Obes. Facts 13, 307–320. doi: 10.1159/000506838 PMC7445574 32369811

[ref26] Cho S. B. Su J. Kuo S. I. Bucholz K. K. Chan G. Edenberg H. J. . (2019). Positive and negative reinforcement are differentially associated with alcohol consumption as a function of alcohol dependence. Psychol. Addict. Behav. 33, 58–68. doi: 10.1037/adb0000436 30667237 PMC6459181

[ref27] Clark M. (2011). Conceptualising addiction: how useful is the construct? Int. J. Hum. Soc. Sci. 1, 55–64.

[ref28] Clemens K. J. Castino M. R. Cornish J. L. Goodchild A. K. Holmes N. M. (2014). Behavioral and neural substrates of habit formation in rats intravenously self-administering nicotine. Neuropsychopharmacology 39, 2584–2593. doi: 10.1038/npp.2014.111 24823947 PMC4207338

[ref29] Crockford D. N. Goodyear B. Edwards J. Quickfall J. El-Guebaly N. (2005). Cue-induced brain activity in pathological gamblers. Biol. Psychiatry 58, 787–795. doi: 10.1016/j.biopsych.2005.04.037 15993856

[ref31] Davis C. (2014). Evolutionary and neuropsychological perspectives on addictive behaviors and addictive substances: relevance to the "food addiction" construct. Subst. Abus. Rehabil. 5, 129–137. doi: 10.2147/SAR.S56835 PMC4270301 25540603

[ref32] De Vries S. K. Meule A. (2016). Food addiction and bulimia nervosa: new data based on the Yale food addiction scale 2.0. Eur. Eat. Disord. Rev. 24, 518–522. doi: 10.1002/erv.2470 27578243

[ref34] Di Chiara G. (1999). Drug addiction as dopamine-dependent associative learning disorder. Eur. J. Pharmacol. 375, 13–30. doi: 10.1016/s0014-2999(99)00372-6 10443561

[ref35] Difeliceantonio A. G. Coppin G. Rigoux L. Edwin Thanarajah S. Dagher A. Tittgemeyer M. . (2018). Supra-additive effects of combining fat and carbohydrate on food reward. Cell Metab. 28:e33. doi: 10.1016/j.cmet.2018.05.018 29909968

[ref36] Ducci F. Goldman D. (2012). The genetic basis of addictive disorders. Psychiatr. Clin. North Am. 35, 495–519. doi: 10.1016/j.psc.2012.03.010 22640768 PMC3506170

[ref37] Espel-Huynh H. M. Muratore A. F. Lowe M. R. (2018). A narrative review of the construct of hedonic hunger and its measurement by the power of food scale. Obes. Sci. Pract. 4, 238–249. doi: 10.1002/osp4.161 29951214 PMC6009994

[ref39] Everitt B. J. Robbins T. W. (2005). Neural systems of reinforcement for drug addiction: from actions to habits to compulsion. Nat. Neurosci. 8, 1481–1489. doi: 10.1038/nn1579 16251991

[ref40] Figee M. Pattij T. Willuhn I. Luigjes J. Van Den Brink W. Goudriaan A. . (2016). Compulsivity in obsessive-compulsive disorder and addictions. Eur. Neuropsychopharmacol. 26, 856–868. doi: 10.1016/j.euroneuro.2015.12.003 26774279

[ref41] Figlewicz D. P. Evans S. B. Murphy J. Hoen M. Baskin D. G. (2003). Expression of receptors for insulin and leptin in the ventral tegmental area/substantia nigra (VTA/SN) of the rat. Brain Res. 964, 107–115. doi: 10.1016/s0006-8993(02)04087-8 12573518

[ref42] Finlayson G. (2017). Food addiction and obesity: unnecessary medicalization of hedonic overeating. Nat. Rev. Endocrinol. 13, 493–498. doi: 10.1038/nrendo.2017.61 28549063

[ref43] Fiorillo C. D. Tobler P. N. Schultz W. (2003). Discrete coding of reward probability and uncertainty by dopamine neurons. Science 299, 1898–1902. doi: 10.1126/science.1077349 12649484

[ref44] Flayelle M. Schimmenti A. Starcevic V. Billieux J. (2022). “The pitfalls of recycling substance-use disorder criteria to diagnose behavioral addictions” in Evaluating the Brain Disease Model of Addiction. eds. Heather N. Field M. Moss A. Satel S. (London: Routledge). doi: 10.4324/9781003032762-34

[ref45] Fletcher P. C. Kenny P. J. (2018). Food addiction: a valid concept? Neuropsychopharmacology 43, 2506–2513. doi: 10.1038/s41386-018-0203-9 30188514 PMC6224546

[ref46] Frank L. E. Nagel S. K. (2017). Addiction and moralization: the role of the underlying model of addiction. Neuroethics 10, 129–139. doi: 10.1007/s12152-017-9307-x 28725284 PMC5486499

[ref47] Garcia-Castro J. Cancela A. Cardaba M. A. M. (2023). Neural cue-reactivity in pathological gambling as evidence for behavioral addiction: a systematic review. Curr Psychol, 42:28026–28037. doi: 10.1007/s12144-022-03915-0 PMC9638381 36373116

[ref48] Gearhardt A. N. Corbin W. R. Brownell K. D. (2009). Preliminary validation of the Yale food addiction scale. Appetite 52, 430–436. doi: 10.1016/j.appet.2008.12.003 19121351

[ref49] Gearhardt A. N. Hebebrand J. (2021). The concept of "food addiction" helps inform the understanding of overeating and obesity: YES. Am. J. Clin. Nutr. 113, 263–267. doi: 10.1093/ajcn/nqaa343 33448279

[ref52] Goldstein R. Z. Volkow N. D. (2011). Dysfunction of the prefrontal cortex in addiction: neuroimaging findings and clinical implications. Nat. Rev. Neurosci. 12, 652–669. doi: 10.1038/nrn3119 22011681 PMC3462342

[ref53] Gordon E. L. Ariel-Donges A. H. Bauman V. Merlo L. J. (2018). What is the evidence for "food addiction?" A Systematic Review. Nutrients 10:477. doi: 10.3390/nu10040477 29649120 PMC5946262

[ref54] Grant J. E. (2006). Understanding and treating kleptomania: new models and new treatments. Isr. J. Psychiatry Relat. Sci. 43, 81–87. 16910369

[ref55] Grant J. E. Brewer J. A. Potenza M. N. (2006). The neurobiology of substance and behavioral addictions. CNS Spectr. 11, 924–930. doi: 10.1017/s109285290001511x 17146406

[ref56] Grant J. E. Kim S. W. (2002). An open-label study of naltrexone in the treatment of kleptomania. J. Clin. Psychiatry 63, 349–356. doi: 10.4088/jcp.v63n0413 12000210

[ref57] Greener M. R. Storr S. J. (2023). Conflicting theories on addiction aetiology and the strengths and limitations of substance use disorder disease modelling. Front. Mol. Neurosci. 16:1166852. doi: 10.3389/fnmol.2023.1166852 37745284 PMC10511750

[ref58] Greenwald A. G. Mcghee D. E. Schwartz J. L. (1998). Measuring individual differences in implicit cognition: the implicit association test. J. Pers. Soc. Psychol. 74, 1464–1480. doi: 10.1037//0022-3514.74.6.1464 9654756

[ref59] Griffiths M. (2005). A 'components' model of addiction within a biopsychosocial framework. J. Subst. Abus. 10, 191–197. doi: 10.1080/14659890500114359

[ref60] Haber S. N. (2014). The place of dopamine in the cortico-basal ganglia circuit. Neuroscience 282, 248–257. doi: 10.1016/j.neuroscience.2014.10.008 25445194 PMC5484174

[ref61] Hagan M. M. Chandler P. C. Wauford P. K. Rybak R. J. Oswald K. D. (2003). The role of palatable food and hunger as trigger factors in an animal model of stress induced binge eating. Int. J. Eat. Disord. 34, 183–197. doi: 10.1002/eat.10168 12898554

[ref62] Hatoum A. S. Colbert S. M. C. Johnson E. C. Huggett S. B. Deak J. D. Pathak G. . (2023). Multivariate genome-wide association meta-analysis of over 1 million subjects identifies loci underlying multiple substance use disorders. Nat. Ment. Health 1, 210–223. doi: 10.1038/s44220-023-00034-y 37250466 PMC10217792

[ref63] Hauck C. Cook B. Ellrott T. (2020). Food addiction, eating addiction and eating disorders. Proc. Nutr. Soc. 79, 103–112. doi: 10.1017/S0029665119001162 31744566

[ref64] Hebebrand J. Albayrak O. Adan R. Antel J. Dieguez C. De Jong J. . (2014). "eating addiction", rather than "food addiction", better captures addictive-like eating behavior. Neurosci. Biobehav. Rev. 47, 295–306. doi: 10.1016/j.neubiorev.2014.08.016 25205078

[ref65] Holmes N. M. Marchand A. R. Coutureau E. (2010). Pavlovian to instrumental transfer: a neurobehavioural perspective. Neurosci. Biobehav. Rev. 34, 1277–1295. doi: 10.1016/j.neubiorev.2010.03.007 20385164

[ref66] Hosseinbor M. Yassini Ardekani S. M. Bakhshani S. Bakhshani S. (2014). Emotional and social loneliness in individuals with and without substance dependence disorder. Int J High Risk Behav Addict 3:e22688. doi: 10.5812/ijhrba.22688 25632385 PMC4295122

[ref68] Insel T. Cuthbert B. Garvey M. Heinssen R. Pine D. S. Quinn K. . (2010). Research domain criteria (RDoC): toward a new classification framework for research on mental disorders. Am. J. Psychiatry 167, 748–751. doi: 10.1176/appi.ajp.2010.09091379 20595427

[ref69] Ioannidis K. Del Giovane C. Tzagarakis C. Solly J. E. Westwood S. J. Parlatini V. . (2025). Pharmacological management of gambling disorder: a systematic review and network meta-analysis. Compr. Psychiatry 137:152566. doi: 10.1016/j.comppsych.2024.152566 39675219

[ref70] James R. J. Tunney R. J. (2017). The need for a behavioural analysis of behavioural addictions. Clin. Psychol. Rev. 52, 69–76. doi: 10.1016/j.cpr.2016.11.010 28013082

[ref71] Kalon E. Hong J. Y. Tobin C. Schulte T. (2016). Psychological and neurobiological correlates of food addiction. Int. Rev. Neurobiol. 129, 85–110. doi: 10.1016/bs.irn.2016.06.003 27503449 PMC5608024

[ref72] Kardefelt-Winther D. Heeren A. Schimmenti A. Van Rooij A. Maurage P. Carras M. . (2017). How can we conceptualize behavioural addiction without pathologizing common behaviours? Addiction 112, 1709–1715. doi: 10.1111/add.13763 28198052 PMC5557689

[ref73] Kayaoglu K. Gokustun K. K. Ay E. (2023). Evaluation of the relationship between food addiction and depression, anxiety, and stress in university students: a cross-sectional survey. J. Child Adolesc. Psychiatr. Nurs. 36, 256–262. doi: 10.1111/jcap.12428 37212020

[ref74] Keys A. Brozek A. Henschel O. Mickelsen H. L. Taylor E. Simonson E. . (1950). The Biology of Human Starvation. Minneapolis: University of Minnesota Press.

[ref76] Khayat A. Yaka R. (2024). Activation of nucleus accumbens projections to the ventral tegmental area alters molecular signaling and neurotransmission in the reward system. Front. Mol. Neurosci. 17:1271654. doi: 10.3389/fnmol.2024.1271654 38528956 PMC10962329

[ref77] Kim H. S. Hodgins D. C. (2018). Component model of addiction treatment: a pragmatic Transdiagnostic treatment model of behavioral and substance addictions. Front. Psych. 9:406. doi: 10.3389/fpsyt.2018.00406 PMC6127248 30233427

[ref78] Koob G. F. Le Moal M. (1997). Drug abuse: hedonic homeostatic dysregulation. Science 278, 52–58. doi: 10.1126/science.278.5335.52 9311926

[ref79] Koob G. F. Le Moal M. (2001). Drug addiction, dysregulation of reward, and allostasis. Neuropsychopharmacology 24, 97–129. doi: 10.1016/S0893-133X(00)00195-0 11120394

[ref80] Kovacs I. Richman M. J. Janka Z. Maraz A. Ando B. (2017). Decision making measured by the Iowa gambling task in alcohol use disorder and gambling disorder: a systematic review and meta-analysis. Drug Alcohol Depend. 181, 152–161. doi: 10.1016/j.drugalcdep.2017.09.023 29055269

[ref81] Kurt E. Yildirim E. Topcuoglu V. (2017). Executive functions of obsessive compulsive disorder and panic disorder patients in comparison to Healty controls. Noro Psikiyatr. Ars. 54, 312–317. doi: 10.5152/npa.2016.14872 29321703 PMC5758073

[ref83] Leehr E. J. Krohmer K. Schag K. Dresler T. Zipfel S. Giel K. E. (2015). Emotion regulation model in binge eating disorder and obesity--a systematic review. Neurosci. Biobehav. Rev. 49, 125–134. doi: 10.1016/j.neubiorev.2014.12.008 25530255

[ref84] Lemieux A. Al'absi M. (2016). Stress psychobiology in the context of addiction medicine: from drugs of abuse to behavioral addictions. Prog. Brain Res. 223, 43–62. doi: 10.1016/bs.pbr.2015.08.001 26806770

[ref85] Levinson C. A. Zerwas S. C. Brosof L. C. Thornton L. M. Strober M. Pivarunas B. . (2019). Associations between dimensions of anorexia nervosa and obsessive-compulsive disorder: an examination of personality and psychological factors in patients with anorexia nervosa. Eur. Eat. Disord. Rev. 27, 161–172. doi: 10.1002/erv.2635 30136346 PMC6913175

[ref86] Limbrick-Oldfield E. H. Mick I. Cocks R. E. Mcgonigle J. Sharman S. P. Goldstone A. P. . (2017). Neural substrates of cue reactivity and craving in gambling disorder. Transl. Psychiatry 7:e992. doi: 10.1038/tp.2016.256 28045460 PMC5545724

[ref87] Lindgren K. P. Neighbors C. Teachman B. A. Wiers R. W. Westgate E. Greenwald A. G. (2013). I drink therefore I am: validating alcohol-related implicit association tests. Psychol. Addict. Behav. 27, 1–13. doi: 10.1037/a0027640 22428863 PMC3604126

[ref88] Luscher C. Ungless M. A. (2006). The mechanistic classification of addictive drugs. PLoS Med. 3:e437. doi: 10.1371/journal.pmed.0030437 17105338 PMC1635740

[ref89] Matar Boumosleh J. Jaalouk D. (2017). Depression, anxiety, and smartphone addiction in university students- a cross sectional study. PLoS One 12:e0182239. doi: 10.1371/journal.pone.0182239 28777828 PMC5544206

[ref90] Mccausland H. C. Gearhardt A. N. Peralta J. M. (2025). A critical evaluation of the terms used to describe foods implicated in addictive-like eating. Curr. Addict. Rep. 12:71. doi: 10.1007/s40429-025-00689-w

[ref92] Meule A. (2015). Back by popular demand: a narrative review on the history of food addiction research. Yale J. Biol. Med. 88, 295–302. doi: 10.5281/ZENODO.48115 26339213 PMC4553650

[ref93] Meule A. Kubler A. (2012). Food cravings in food addiction: the distinct role of positive reinforcement. Eat. Behav. 13, 252–255. doi: 10.1016/j.eatbeh.2012.02.001 22664405

[ref94] Miedl S. F. Buchel C. Peters J. (2014). Cue-induced craving increases impulsivity via changes in striatal value signals in problem gamblers. J. Neurosci. 34, 4750–4755. doi: 10.1523/JNEUROSCI.5020-13.2014 24672019 PMC6608125

[ref95] Mond J. M. Robertson-Smith G. Vetere A. (2006). Stigma and eating disorders: is there evidence of negative attitudes towards anorexia nervosa among women in the community? J. Ment. Health 15, 519–532. doi: 10.1080/09638230600902559

[ref96] Morris L. S. Voon V. (2016). Dimensionality of cognitions in behavioral addiction. Curr. Behav. Neurosci. Rep. 3, 49–57. doi: 10.1007/s40473-016-0068-3 27034915 PMC4769313

[ref97] Niu G. F. Sun X. J. Subrahmanyam K. Kong F. C. Tian Y. Zhou Z. K. (2016). Cue-induced craving for internet among internet addicts. Addict. Behav. 62, 1–5. doi: 10.1016/j.addbeh.2016.06.012 27305097

[ref98] Nutt D. J. Lingford-Hughes A. Erritzoe D. Stokes P. R. (2015). The dopamine theory of addiction: 40 years of highs and lows. Nat. Rev. Neurosci. 16, 305–312. doi: 10.1038/nrn3939 25873042

[ref99] Olmstead M. C. Lafond M. V. Everitt B. J. Dickinson A. (2001). Cocaine seeking by rats is a goal-directed action. Behav. Neurosci. 115, 394–402. doi: 10.1037/0735-7044.115.2.394 11345964

[ref100] Panny B. Price R. B. Wears A. Ahmari S. E. (2024). Altered neural activity during negative reinforcement in people with obsessive-compulsive disorder. Cogn. Ther. Res. 48, 737–748. doi: 10.1007/s10608-024-10475-z

[ref101] Parylak S. L. Koob G. F. Zorrilla E. P. (2011). The dark side of food addiction. Physiol. Behav. 104, 149–156. doi: 10.1016/j.physbeh.2011.04.063 21557958 PMC3304465

[ref102] Passeri A. Municchi D. Cavalieri G. Babicola L. Ventura R. Di Segni M. (2023). Linking drug and food addiction: an overview of the shared neural circuits and behavioral phenotype. Front. Behav. Neurosci. 17:1240748. doi: 10.3389/fnbeh.2023.1240748 37767338 PMC10520727

[ref103] Pelchat M. L. (2002). Of human bondage: food craving, obsession, compulsion, and addiction. Physiol. Behav. 76, 347–352. doi: 10.1016/s0031-9384(02)00757-6 12117571

[ref104] Perales J. C. King D. L. Navas J. F. Schimmenti A. Sescousse G. Starcevic V. . (2020). Learning to lose control: a process-based account of behavioral addiction. Neurosci. Biobehav. Rev. 108, 771–780. doi: 10.1016/j.neubiorev.2019.12.025 31846653

[ref105] Perera M. P. N. Mallawaarachchi S. Bailey N. W. Murphy O. W. Fitzgerald P. B. (2023). Obsessive-compulsive disorder (OCD) is associated with increased electroencephalographic (EEG) delta and theta oscillatory power but reduced delta connectivity. J. Psychiatr. Res. 163, 310–317. doi: 10.1016/j.jpsychires.2023.05.026 37245318

[ref106] Petry N. M. Zajac K. Ginley M. K. (2018). Behavioral addictions as mental disorders: to be or not to be? Annu. Rev. Clin. Psychol. 14, 399–423. doi: 10.1146/annurev-clinpsy-032816-045120 29734827 PMC5992581

[ref108] Randolph T. G. (1956). The descriptive features of food addiction; addictive eating and drinking. Q. J. Stud. Alcohol. 17, 198–224. doi: 10.15288/QJSA.1956.17.198 13336254

[ref110] Robbins T. W. Clark L. (2015). Behavioral addictions. Curr. Opin. Neurobiol. 30, 66–72. doi: 10.1016/j.conb.2014.09.005 25262209

[ref111] Robbins T. W. Gillan C. M. Smith D. G. De Wit S. Ersche K. D. (2012). Neurocognitive endophenotypes of impulsivity and compulsivity: towards dimensional psychiatry. Trends Cogn. Sci. 16, 81–91. doi: 10.1016/j.tics.2011.11.009 22155014

[ref112] Robinson T. E. Berridge K. C. (2008). Review. The incentive sensitization theory of addiction: some current issues. Philos. Trans. R. Soc. Lond. Ser. B Biol. Sci. 363, 3137–3146. doi: 10.1098/rstb.2008.0093 18640920 PMC2607325

[ref113] Roh D. Bhang S. Y. Choi J. S. Kweon Y. S. Lee S. K. Potenza M. N. (2018). The validation of implicit association test measures for smartphone and internet addiction in at-risk children and adolescents. J. Behav. Addict. 7, 79–87. doi: 10.1556/2006.7.2018.02 29383939 PMC6035023

[ref114] Ross C. A. Margolis R. L. (2019). Research domain criteria: strengths, weaknesses, and potential alternatives for future psychiatric research. Mol. Neuropsych. 5, 218–236. doi: 10.1159/000501797 PMC6873013 31768375

[ref115] Sanchez-Roige S. Palmer A. A. (2020). Emerging phenotyping strategies will advance our understanding of psychiatric genetics. Nat. Neurosci. 23, 475–480. doi: 10.1038/s41593-020-0609-7 32231337 PMC9200410

[ref116] Schiestl E. T. Gearhardt A. N. (2018). Preliminary validation of the Yale food addiction scale for children 2.0: a dimensional approach to scoring. Eur. Eat. Disord. Rev. 26, 605–617. doi: 10.1002/erv.2648 30334311 PMC6231957

[ref117] Schreiber L. R. Odlaug B. L. Grant J. E. (2013). The overlap between binge eating disorder and substance use disorders: diagnosis and neurobiology. J. Behav. Addict. 2, 191–198. doi: 10.1556/JBA.2.2013.015 25215200 PMC4154572

[ref118] Schulte E. M. Avena N. M. Gearhardt A. N. (2015). Which foods may be addictive? The roles of processing, fat content, and glycemic load. PLoS One 10:e0117959. doi: 10.1371/journal.pone.0117959 25692302 PMC4334652

[ref119] Schulte E. M. Gearhardt A. N. (2017). Development of the modified Yale food addiction scale version 2.0. Eur. Eat. Disord. Rev. 25, 302–308. doi: 10.1002/erv.2515 28370722

[ref121] Singh D. Saadabadi A. (2025). "naltrexone," in *StatPearls*. (Treasure Island (FL) ineligible companies. Disclosure: Abdolreza Saadabadi declares no relevant financial relationships with ineligible companies).

[ref122] Sinha R. (2008). Chronic stress, drug use, and vulnerability to addiction. Ann. N. Y. Acad. Sci. 1141, 105–130. doi: 10.1196/annals.1441.030 18991954 PMC2732004

[ref123] Sjoerds Z. De Wit S. Van Den Brink W. Robbins T. W. Beekman A. T. Penninx B. W. . (2013). Behavioral and neuroimaging evidence for overreliance on habit learning in alcohol-dependent patients. Transl. Psychiatry 3:e337. doi: 10.1038/tp.2013.107 24346135 PMC4030326

[ref125] Solomon R. L. Corbit J. D. (1978). An opponent-process theory of motivation. Am. Econ. Rev. 68, 12–24. doi: 10.1037/h0036128

[ref126] Stroop J. R. (1935). Studies of interference in serial verbal reactions. J. Exp. Psychol. 18, 643–662. doi: 10.1037/h0054651

[ref127] Sussman S. Sussman A. N. (2011). Considering the definition of addiction. Int. J. Environ. Res. Public Health 8, 4025–4038. doi: 10.3390/ijerph8104025 22073026 PMC3210595

[ref128] Tadayonnejad R. Majid D. A. Tsolaki E. Rane R. Wang H. Moody T. D. . (2022). Mesolimbic neurobehavioral mechanisms of reward motivation in anorexia nervosa: a multimodal imaging study. Front. Psych. 13:806327. doi: 10.3389/fpsyt.2022.806327 PMC8934777 35321230

[ref129] Tahir T. Wong M. M. Maaz M. Naufal R. Tahir R. Naidoo Y. (2022). Pharmacotherapy of impulse control disorders: a systematic review. Psychiatry Res. 311:114499. doi: 10.1016/j.psychres.2022.114499 35305343

[ref130] Torrens M. Fonseca F. Mateu G. Farre M. (2005). Efficacy of antidepressants in substance use disorders with and without comorbid depression. A systematic review and meta-analysis. Drug Alcohol Depend. 78, 1–22. doi: 10.1016/j.drugalcdep.2004.09.004 15769553

[ref131] Trotzke P. Starcke K. Pedersen A. Brand M. (2014). Cue-induced craving in pathological buying: empirical evidence and clinical implications. Psychosom. Med. 76, 694–700. doi: 10.1097/PSY.0000000000000126 25393125

[ref133] Uhl G. R. Koob G. F. Cable J. (2019). The neurobiology of addiction. Ann. N. Y. Acad. Sci. 1451, 5–28. doi: 10.1111/nyas.13989 30644552 PMC6767400

[ref135] Vainik U. Misic B. Zeighami Y. Michaud A. Mottus R. Dagher A. (2020). Obesity has limited behavioural overlap with addiction and psychiatric phenotypes. Nat. Hum. Behav. 4, 27–35. doi: 10.1038/s41562-019-0752-x 31659319

[ref136] Vasiliu O. (2021). Current status of evidence for a new diagnosis: food addiction-a literature review. Front. Psych. 12:824936. doi: 10.3389/fpsyt.2021.824936 PMC8784968 35082706

[ref137] Vezina P. Leyton M. (2009). Conditioned cues and the expression of stimulant sensitization in animals and humans. Neuropharmacology 56, 160–168. doi: 10.1016/j.neuropharm.2008.06.070 18657553 PMC2635339

[ref138] Volkow N. D. Baler R. D. (2014). Addiction science: uncovering neurobiological complexity. Neuropharmacology 76 Pt B, 235–249. doi: 10.1016/j.neuropharm.2013.05.007 23688927 PMC3818510

[ref139] Volkow N. D. Koob G. F. Mclellan A. T. (2016). Neurobiologic advances from the brain disease model of addiction. N. Engl. J. Med. 374, 363–371. doi: 10.1056/NEJMra1511480 26816013 PMC6135257

[ref140] Volkow N. D. Wang G. J. Fowler J. S. Tomasi D. Telang F. (2011). Addiction: beyond dopamine reward circuitry. Proc. Natl. Acad. Sci. USA 108, 15037–15042. doi: 10.1073/pnas.1010654108 21402948 PMC3174598

[ref141] Volkow N. D. Wise R. A. Baler R. (2017). The dopamine motive system: implications for drug and food addiction. Nat. Rev. Neurosci. 18, 741–752. doi: 10.1038/nrn.2017.130 29142296

[ref142] Vollstadt-Klein S. Wichert S. Rabinstein J. Buhler M. Klein O. Ende G. . (2010). Initial, habitual and compulsive alcohol use is characterized by a shift of cue processing from ventral to dorsal striatum. Addiction 105, 1741–1749. doi: 10.1111/j.1360-0443.2010.03022.x 20670348

[ref143] Wang G. J. Geliebter A. Volkow N. D. Telang F. W. Logan J. Jayne M. C. . (2011). Enhanced striatal dopamine release during food stimulation in binge eating disorder. Obesity (Silver Spring) 19, 1601–1608. doi: 10.1038/oby.2011.27 21350434 PMC3144277

[ref144] Wang G. J. Volkow N. D. Logan J. Pappas N. R. Wong C. T. Zhu W. . (2001). Brain dopamine and obesity. Lancet 357, 354–357. doi: 10.1016/s0140-6736(00)03643-6 11210998

[ref145] Weatherly J. N. Mari K. Montes K. S. (2012). Gambling in a laboratory setting: a comparison of gambling for positive reinforcement versus as a potential escape. Anal. Gambl. Behav. 6:4.

[ref146] Wegmann E. Stodt B. Brand M. (2018). Cue-induced craving in internet-communication disorder using visual and auditory cues in a cue-reactivity paradigm. Addict. Res. Theor. 26, 306–314. doi: 10.1080/16066359.2017.1367385

[ref147] Wei Z. Zhang X. (2017). Similarities and differences in diagnostic criterion. Adv. Exp. Med. Biol. 1010, 105–132. doi: 10.1007/978-981-10-5562-1_7 29098671

[ref148] Weil A. T. (1978). Coca leaf as a therapeutic agent. Am. J. Drug Alcohol Abuse 5, 75–86. doi: 10.3109/00952997809029262 696708

[ref149] West R. Marsden J. Hastings J. (2019). Addiction theories and constructs: a new series. Addiction 114, 955–956. doi: 10.1111/add.14554 30644145

[ref150] Wise R. A. Robble M. A. (2020). Dopamine and addiction. Annu. Rev. Psychol. 71, 79–106. doi: 10.1038/nrn1406 31905114

[ref151] Wiss D. A. Avena N. M. (2020). “Food addiction, binge eating, and the role of dietary restraint: converging evidence from animal and human studies” in Binge Eating. eds. Frank G. Berner L. (Cham: Springer). doi: 10.1007/978-3-030-43562-2_14

[ref152] Witt A. A. Lowe M. R. (2014). Hedonic hunger and binge eating among women with eating disorders. Int. J. Eat. Disord. 47, 273–280. doi: 10.1002/eat.22171 24014479

[ref153] World Health Organization (1992). The ICD-10 Classification of Mental and Behavioural Disorders: Clinical Descriptions and Diagnostic Guidelines. Geneva: World Health Organization.

[ref154] World Health Organization (2018). International Classification of Diseases for Mortality and Morbidity Statistics (11th revision). Geneva: World Health Organization.

[ref155] Yap J. J. Miczek K. A. (2008). Stress and rodent models of drug addiction: role of VTA-Accumbens-PFC-amygdala circuit. Drug Discov. Today Dis. Models 5, 259–270. doi: 10.1016/j.ddmod.2009.03.010 20016773 PMC2794209

[ref156] Yucel M. Oldenhof E. Ahmed S. H. Belin D. Billieux J. Bowden-Jones H. . (2019). A transdiagnostic dimensional approach towards a neuropsychological assessment for addiction: an international Delphi consensus study. Addiction 114, 1095–1109. doi: 10.1111/add.14424 30133930 PMC6386631

[ref157] Zapata A. Minney V. L. Shippenberg T. S. (2010). Shift from goal-directed to habitual cocaine seeking after prolonged experience in rats. J. Neurosci. 30, 15457–15463. doi: 10.1523/JNEUROSCI.4072-10.2010 21084602 PMC3073559

[ref158] Zhang J. T. Yao Y. W. Potenza M. N. Xia C. C. Lan J. Liu L. . (2016). Effects of craving behavioral intervention on neural substrates of cue-induced craving in internet gaming disorder. Neuroimage Clin. 12, 591–599. doi: 10.1016/j.nicl.2016.09.004 27699148 PMC5035334

